# Multiple Mechanisms Required to Predict Grass Community Composition

**DOI:** 10.1111/ele.70358

**Published:** 2026-04-12

**Authors:** Jane A. Catford, Laura J. Graham, Harry E. R. Shepherd, Cindy E. Hauser, Nicola T. Munro, Brendan A. Wintle, John G. Donohue, David Tilman, Adam T. Clark

**Affiliations:** ^1^ Department of Geography King’s College London London UK; ^2^ Fenner School of Environment and Society The Australian National University Canberra ACT Australia; ^3^ Melbourne Biodiversity Institute, School of Agriculture, Food and Ecosystem Sciences The University of Melbourne Parkville Victoria Australia; ^4^ School of Geography, Earth and Environmental Sciences University of Birmingham Birmingham UK; ^5^ Biodiversity, Ecology and Conservation Group International Institute for Applied Systems Analysis Vienna Austria; ^6^ Animal and Plant Health Agency Surrey UK; ^7^ Department of Ecology, Evolution and Behavior University of Minnesota Saint Paul Minnesota USA; ^8^ Bren School of Environmental Science and Management University of California Santa Barbara California USA; ^9^ Department of Biology University of Graz Graz Austria

**Keywords:** biodiversity change, community assembly, community ecology theory, grass species, grassland experiment, mechanistic niche model, metacommunity dynamics, plant community composition, quantitative predictions, species coexistence

## Abstract

Accurate prediction of community assembly is a central goal in ecology but is challenging because assembly is governed by numerous mechanisms. Few theoretical models explicitly incorporate or test multiple mechanisms at once. We empirically tested the predictive performance of a plant community assembly model built using all possible combinations of four ‘mechanisms’ (soil resource competition, dispersal and colonisation, spatiotemporal niche differentiation, population growth rates) and 11 underlying ‘attributes’ based on measured traits (e.g., fecundity, phenology). The full model accurately predicted out‐of‐sample biomass observations of five grasses sown in mixture along a soil nitrogen gradient (overall *R* ^2^ = 0.65). Alternative model variants, parameterised using subsets of the mechanisms and their nested attributes, still retained high explanatory power if the model included at least three of the four mechanisms. Our results suggest that plant community composition is determined by simultaneous effects of multiple mechanisms, and simpler theories have much lower predictive abilities.

## Graphical abstract

In efforts to improve the predictive understanding of plant community assembly, this study evaluates how well different combinations of ecological mechanisms predict plant community composition. Our multi‐mechanism niche model could reliably predict out‐of‐sample biomass observations of five grasses sown in mixture along a soil nitrogen gradient when the model incorporated at least three of four key mechanisms, though the full model performed best (*R* ^2^ = 0.65). Our results suggest that plant community composition is determined by simultaneous effects of multiple mechanisms, and simpler theories have much lower predictive abilities.

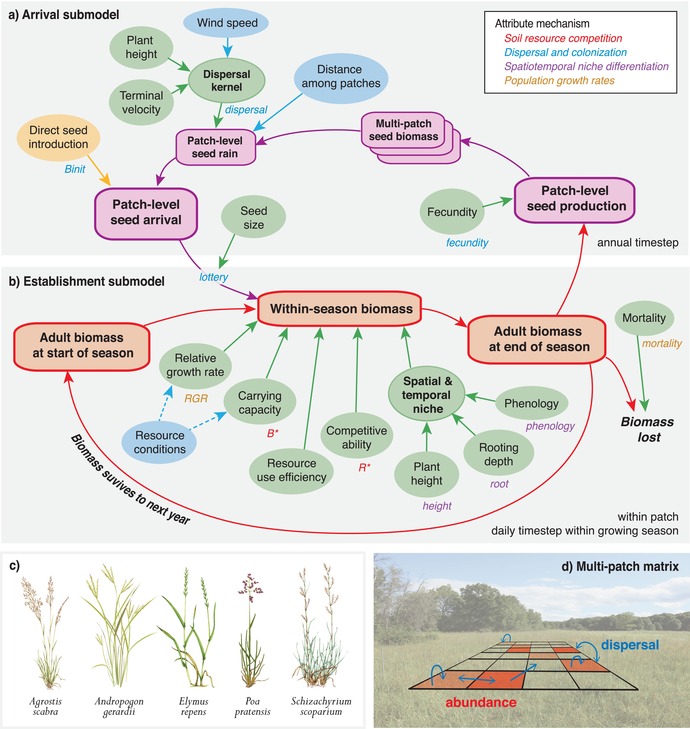

## 1 Introduction

Predicting community composition has long been a goal in ecology (Lee et al. 2023), so numerous mechanisms of community assembly have been proposed (Vellend 2016; Leibold and Chase 2018). Competition for one or more limiting resources forms the basis of numerous theories in plant ecology. Resource competition is sometimes paired with other mechanisms, including colonisation (reflecting a tradeoff between species characteristics associated with competitive and colonisation abilities, Tilman 1994) and spatiotemporal niche differentiation (where plants can effectively reduce competition by growing at different times or in different spaces, Wright 2002; Levine, Levine, and Pacala 2025). Stochasticity, demographic rates and environmental suitability have also been incorporated into competition models, recognising their importance in determining community composition (i.e., the identities and abundances of species in an ecological assemblage, Tilman 2004; Bowler et al. 2022). Many community ecology models do not include competition though (Vellend 2016). For example, community assembly can be predicted from spatial dynamics and mass effects where individuals disperse from patches with large populations to patches with small populations (Waller et al. 2018).

Despite broad recognition that community assembly is multifaceted (Vellend 2016; Leibold and Chase 2018), few theoretical models in community ecology consider multiple mechanisms at once (Thompson et al. 2020; Lerch et al. 2023). A historical preference for simpler models likely reflects the trade‐off among precision, realism and generality (Fordham et al. 2018), at least in part. However, by preferencing simpler models that tend to focus on one mechanism at a time, correlations and complementarity among mechanisms could result in certain mechanisms being over‐ or under‐acknowledged. Such bias could limit fundamental understanding but also affect management actions. For example, the historical research focus on competition over facilitation (Lortie and Callaway 2009) may have biased vegetation restoration to prioritize weed control over the introduction of mutualists such as soil inocula (Benayas et al. 2009; Wubs et al. 2016).

Mechanistic models of plant community composition are needed for explanatory (or corroboratory) prediction, which advances theory, and for anticipatory prediction (or forecasting, Mouquet et al. 2015). Forecasting community composition has always been challenging but is becoming increasingly difficult because of the rise in novel ecological conditions associated with the Anthropocene (Tatsumi et al. 2021; Pili et al. 2024). To date, most predictive models of community composition have relied on statistical pattern‐matching approaches, which fit patterns to data (Blonder et al. 2024). At least historically, these so‐called ‘phenomenological models’ tend to perform poorly outside of the range of conditions under which they were parameterised and can suffer from overfitting (Norberg et al. 2019). While mechanism‐based models are better equipped to extrapolate to new combinations of species and environmental conditions (Briscoe et al. 2019) and avoid issues of overfitting, traditional mechanism‐based models are typically too demanding regarding input data (Hartig et al. 2012; Blonder et al. 2023), occur at the wrong scale (Clark et al. 2019), and are unable to deal with the number and novel combinations of taxa being mixed (Catford et al. 2018). Mechanistic niche models that are built using information about individual taxa and account for metacommunity dynamics seem most promising for forecasting community composition in the Anthropocene.

**FIGURE 1:**
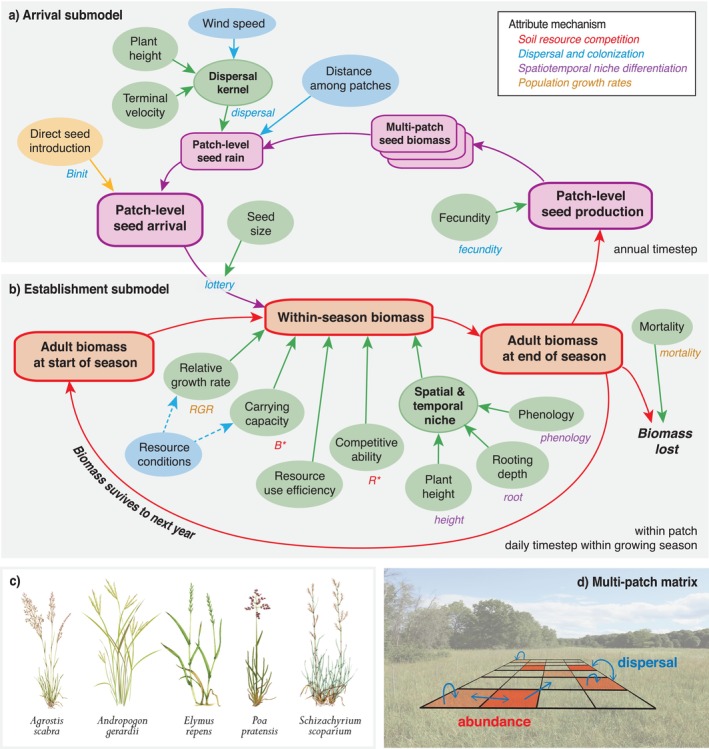
Representation of multi‐mechanism niche model for quantitatively predicting grassland plant community composition showing (a) arrival and (b) establishment submodels, tested with (c) five species that were experimentally added to a (d) matrix of patches. The submodel schematics show attributes affecting aboveground biomass (abundance) of plant species as (a) propagules in a single patch (community) set within multiple patches (metacommunity) and (b) adult plants in a single patch. A lottery function links the two submodels. Variables in bold are predicted by the model; parameters in regular font require input data. Balloon colours indicate data input type: green, species‐specific characteristics of all plant species in the (meta) community; blue, abiotic and geographic conditions; yellow, human addition of plant propagules. Dashed arrows indicate parameters that are modulated based on local resource conditions (i.e., patch soil N). Adult biomass across multiple patches in the metacommunity affects seed rain. The 11 attributes that we switch on/off in the model are italicised, and the mechanism that they represent is noted by font colour (Table 1). Submodel timesteps are noted (daily or annual). Seed size is measured and modelled as mass per individual seed, but we call it seed size to avoid confusion with seed biomass (i.e., biomass of all seeds). (c) *Agrostis, Elymus* and *Schizachyrium* illustrations by Lizzie Harper; *Andropogon* and *Poa* illustrations by Tracey Saxby; (d) photo by Jane Catford.

In this study, we aim to quantitatively predict the composition of grass species mixtures under different environmental conditions and to determine the relative importance of different mechanisms driving their composition. To achieve this, we built on existing theoretical approaches to create a hierarchical multi‐mechanism niche model for predicting plant community composition (Figure 1). Our mechanistic niche model incorporates local and regional dynamics and stochasticity and is based on four key ‘mechanisms’, which can interact: (i) soil resource competition, (ii) local dispersal and colonisation, (iii) spatiotemporal niche differentiation and (iv) population growth rates. These four mechanisms can be described using attributes such as species' phenology, fecundity and resource requirements, which may serve as independent variables in a model (Figure 1). We used 11 attributes to describe the four mechanisms (Table 1), which we factorially switched on and off to examine their relative importance (Gotelli et al. 2009) and how data availability affected model performance, meeting a recognised need (Briscoe et al. 2019). The model is designed so that all species perform identically when all attributes and mechanisms are switched off (Table 1, Appendix S1). We quantified the out‐of‐sample performance of our model by applying it to a grass biodiversity experiment conducted at Cedar Creek Ecosystem Science Reserve (henceforth Cedar Creek) in Minnesota, USA. The experiment involved sowing five common perennial grass species in 1‐, 2‐, 3‐ and 5‐species mixtures along a gradient of soil nitrogen, which has been identified as a key limiting resource at Cedar Creek (Wedin and Tilman 1993). We parameterised the model using independent data on species traits (Figure 1) and evaluated the model by comparing its out‐of‐sample predictions with observed aboveground biomass after 6 years of growth.

**TABLE 1:** Description of 11 attributes used in the multi‐mechanism niche model, including their overarching mechanism, data source and how each attribute is ‘switched off’ in the model. Through these attributes, our model captures—to varying degrees—selection, dispersal and drift (sensu Vellend 2016). Attributes were grouped based on the mechanism to which they contribute or underlie. ‘Population growth rates’ (Mechanism 4) can function independently of the other mechanisms, but is not a mechanism per se. However, for simplicity, we call it a mechanism in accordance with the hierarchical structure of our model. For most attributes, switching them off turns off species‐level differences such that all species take the same value.

Attribute	Explanation	Data source	Effect when switched off
*Mechanism 1: Resource competition*			
R* (competitive ability for soil nitrogen)	Determines competitive rank of species (based on competitive ability for soil nitrogen, the limiting resource at Cedar Creek), which affects competitive hierarchy among adult plants.	Mean residual soil nitrate concentration, calculated from all monocultures at Cedar Creek, as reported in Clark et al. (2018). A single value is used for each species as there was insufficient data to quantify R* values for specific soil N levels.	All species take the same R* by setting intraspecific interaction strength to equal interspecific interaction strength.
B* (carrying capacity)	Species biomass at equilibrium when grown in monoculture. Effectively determines species' carrying capacity based on soil N (assuming no transgressive overyielding when grown in polyculture).	Initial estimates were taken from species' monoculture plots in E026 (year 6 through year 9 excluding block 5, which was fertilised). For each species, we fit a mixed effects model, tracking log‐transformed biomass as a function of log‐transformed total soil N concentration, such that the intercept varied by species, whereas the slope of the regression was constant across species. We then used predictions from this model to estimate B* for each species in each plot in the experiment (including plots that were not monocultures).	All species take the same B* based on the average of the five species.
*Mechanism 2: Dispersal and colonisation*			
*B_init_* (initial biomass)	Species‐level biomass added by humans from an external source. Determines biomass at the start of the first growing season (i.e., *b*_*i*_(*y*_0_)).	Species initial biomass is set based on the experimental seeding rates for each plot that were used to start the experiment, as reported in Tilman and Wedin (1991) and Wedin and Tilman (1993).	All species and plots take the same *B_init_*, based on the average seeding rate across all species that were sown in plot *j*.
Fecundity	Reproduction rate. Proportion of biomass that goes towards seed production each year; the portion is ‘safe’ and does not experience mortality.	Measured from monocultures at Cedar Creek.	All species take the same fecundity based on the average of the five species.
Dispersal	Seeds can disperse among plots following the dispersal kernel.	Calculated in model, as specified in methods and R code.	Turns off between‐plot dispersal, such that seeds must stay in the same plot in which they were produced (effectively isolating plots from one another).
Lottery	To germinate, seeds that are produced by each species compete via a lottery function for unused resources in each plot following random uniform draws from the full seed pool. Provides a source of stochasticity, enabling ecological drift (sensu Vellend 2016).	Calculated in model, as specified in methods and R code.	Turns off lottery function, such that unused resources are divided deterministically among species, in proportion to their seed biomass. When there is high resource availability relative to species resource needs (*q*), the random and deterministic draws would produce similar results. When resources are limited, the lottery function can be more influential.
*Mechanism 3: Spatiotemporal niche differentiation*			
Root (maxima rooting depth)	Maximum rooting depth for each species. Determines belowground spatial overlap of species.	Information was compiled at the species level from the US Federal Fire Effects Information System (Species Reviews) (Uchytil 1988; Snyder 1992; Uchytil 1993; Steinberg 2002), except for *Agrostis*, which was sourced from USDA Plants database (https://plants.usda.gov/plant‐profile/AGSC5) and (Weaver 1926) (https://soilandhealth.org/wp‐content/uploads/01aglibrary/010139fieldcroproots/010139ch11.html).	All species take the same rooting depth based on the average of the five species.
Height	Height of plants above ground level when growing in polyculture. Determines height at which plants release seed and extent of aboveground spatial overlap.	Data from Sullivan et al. (2018) based on plants growing in polyculture.	All species take the same plant height based on the average of the five species. Switch only turns off aboveground niche partitioning — it does not change seed dispersal dynamics.
Phenology	Months during which species grow, specified as start month and end month. Determines temporal overlap of species; matrix used to determine extent of overlap.	Information was compiled from the US Federal Fire Effects Information System (Species Reviews) (Uchytil 1988; Matthews 1992; Snyder 1992; Uchytil 1993; Steinberg 2002), supplemented with the following sources for *Poa* (https://aggie‐hort.tamu.edu/plantanswers/turf/publications/Bluegrass.html, (Global Invasive Species Database 2025)) and *Agrostis* ((Tercek & Whitbeck 2004); https://en.hortipedia.com/Agrostis_scabra; https://www.wildflower.org/plants/result.php?id_plant=AGSC5). Data based on plants growing in polyculture.	All species take the same phenology so temporal overlap is identical across all species pairs.
*Mechanism 4: Population growth rates*			
Mortality	Proportion of adult biomass that doesn't go towards reproduction is exposed to mortality from one growing season to the next; effectively reduces biomass from *B* to *b*	Values for *Schizachyrium* and *Andropogon* are minimum mortality rates as measured by Lauenroth and Adler (2008); mortality rates of the other three species estimated via interpolation, based on a PCA across all available trait values (i.e., we fit a PCA across all observed traits, with missing trait values set to the community‐level mean, and then used the resulting PCA to estimate missing trait values based on the full relationship observed across all traits and species).	All species take the same mortality based on the average of the five species.
Relative growth rate (*RGR*)	Determines rate at which each g of plant biomass (whether seed or adult) grows per day, dependent on plot soil N	Initial estimates for each species were made using the mean value per species taken from across all monocultures at Cedar Creek. These values were then adjusted using information from Tilman and Cowan (1989), which examined how *RGR* varied with soil N. To do so, we fit a mixed effects model of *RGR* vs total soil N across all 8 species included in the Tilman and Cowan (1989) experiment (including Schiz*achyrium, Poa, Agrostis*), such that both the intercept and slope of the model varied by species. For species not included in the Tilman and Cowan (1989) paper, we used the fixed effects (‘population‐level’) estimate for the slope. Otherwise, we used the species‐specific random effect.	All species take the same RGR based on the average of the five species.

## 2 Materials and Methods

### 2.1 Model Structure and Development

We designed and constructed our model using theory derived from movement (Bullock et al. 2017; Sullivan et al. 2018), (meta‐)population (Pacala and Silander Jr. 1985; Hanski 1991), (meta‐)community (Tilman 1982; Tilman 1994, 2004; Chesson and Kuang 2008; Clark et al. 2018; Leibold and Chase 2018; Wandrag et al. 2019; Thompson et al. 2020) and invasion ecology (Catford et al. 2009; Leung et al. 2012; Gilbert and Levine 2013; Catford et al. 2018). The model aims to predict occupancy and abundance of plant species populations in an array of patches and is split, for logistical convenience, into two submodels—one that predicts arrival of species to a patch and one that predicts the establishment and subsequent growth of species' populations within patches (Figure 1). The submodels are linked via a lottery function and are specified as three linked sets of mathematical equations, implemented for modelling in R (version 4.1.0; R Core Team 2025). We describe the general procedure for the model below. Further information is provided in Appendix S1 and full details are provided in the ‘main_function.R’ script (https://github.com/laurajanegraham/simulateCoexistence), which is the primary function used to run our model and includes links to required data.

In the model, aboveground biomass is simulated at two temporal scales (Figure 1):

- *b*_*ij*_(*y*), which tracks annual changes in biomass of species *i* in patch *j* at the start of the growing season in year *y*; and
- *B*_*ij*_(*y*, *t*), which tracks within‐growing‐season growth, where *t* is an index for growing season day, ranging from *t =* 0 (start of season) to *t =* 160 (end of season), roughly matching the length of growing season at Cedar Creek.

Note that *b*_*ij*_(*y*) = *B*_*ij*_(*y*, *t =* 0), but—for clarity—we use separate variables for the annual versus daily timesteps.

The *arrival submodel* considers introductions of plant biomass for species *i* at the start of growing season *y* into patch *j*. Initial biomass in year 0, *b*_*ij*_(*y =* 0), is based on seed biomass added directly by humans (*B_init_*) from an external source and, in later years (*y* > 0), seeds dispersed from local plant populations that were produced in the previous growing season (‘seed rain’). Seed rain is determined by a spatially explicit function of the total reproductive (seed) biomass produced by species *i* across the multi‐patch matrix. The probability density function describing the likelihood that a seed of species *i* disperses distance *d* from a focal patch (or site), *ps*_*i*_(*d*), is computed following the Wald Analytical Long‐distance Dispersal model (Sullivan et al. 2018):

$${\mathrm{ps}}_{i}\left(d\right)={\left(\frac{\lambda_{i}}{\text{2π}d^{3}}\right)}^{\left(1/2\right)}\;\exp\left(\frac{-\lambda_{i}{\left(d-\mu_{i}\right)}^{2}}{2{\mu_{i}}^{2}\;d}\right)$$

where *λ*_*i*_ and *μ*_*i*_ are fitted constants, describing the dispersion and mean of the Wald kernel, calculated as:

$$\lambda_{i}=\frac{{h_{i}}^{2}}{\sigma^{2}}$$

$$\mu_{i}=\frac{h_{i}w}{v_{i}}$$

where *h*_*i*_ is height of seed release, *w* is average wind velocity, *v*_*i*_ is seed terminal velocity and *σ* ^2^ = 0.3 describes the impacts of wind flow above the canopy.

The fraction of seed biomass that disperses between patches, or remains within a particular patch, is then calculated by simulating dispersal of a large number of seeds (2 × 10^4^ per species and patch), where distance travelled by each seed is determined by *ps*_*i*_(*d*), and the dispersal direction is chosen from a random uniform distribution of 0 to 360°. This result is then stored in a three‐dimensional array *pd*, where *pd*_*i,j,k*_ describes the fraction of seeds of species *i* that disperse from patch *j* to patch *k*. The biomass of seed rain that reaches each patch from the multi‐patch matrix in year y, *D*_*i*_(*y*), is thus determined by: the biomass of species *i* in each component patch at the end of the last growing season, *B*_*ij*_(*y‐1, t =* 160); multiplied by the fraction of that biomass that is allocated to reproduction, *f*_*i*_; multiplied by the total fraction of seed output that lands in the focal patch from each component patch *pd*_*i,j,k*_.

The *establishment submodel* predicts patch‐level dynamics based on competition for soil nitrogen (Tilman 1982; Clark et al. 2018), modified to account for spatiotemporal niche differentiation among species. Following model initialisation, biomass at the start of each subsequent growing season *y* > 0 is determined by:

$$b_{\mathrm{ij}}\left(y\right)=B_{\mathrm{ij}}\left(y-1,t=160\right)\times \left(1-m_{i}\right)\times \left(1-f_{i}\right)$$

where *m*_*i*_ represents the biomass lost to respiration and mortality over the winter, and *f*_*i*_ is the biomass allocated to seeds.

Dynamics within the growing season are modelled using a continuous‐time resource competition model, modified to account for effects of spatial and temporal niche overlap among species, which represents spatiotemporal niche differentiation. Spatiotemporal niche differentiation reduces competitive impacts among species, proportional to species' belowground, aboveground and phenological overlap. Spatiotemporal niche differentiation can be asymmetric reflecting differences in species' morphology and phenology. For example, if species A roots to 50 cm and species B roots to 100 cm, the competitive impact of species B on A would be weighted at 100% because 100% of species A roots would be affected by species B roots, whereas the impact of A on B would be reduced by 50%.

Height and root overlap are calculated as the fraction of total soil or aboveground space occupied by species *i* that is also occupied by a different species *i’*, i.e.,:

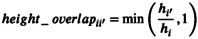




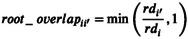

where *h* and *rd* are plant species height and maximum rooting depth, respectively. Total *spatial_overlap*_*ii’*_ is then calculated as the arithmetic mean of these two quantities. Phenological overlap is calculated as the total fraction of the growing season of species *i* when species *i’* is also active, i.e.,:

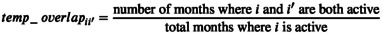

Total overlap is then calculated as:

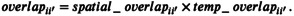

Finally, community dynamics within each growing season follow the growth model of Clark et al. (2018):

$$\begin{array}{c} \frac{dB_{ij}\left(y,t\right)}{dt}=RGR_{ij}\times \left(B_{ij}^{*}-B_{ij}\left(y,t\right)-\sum_{\forall R_{i^{\prime }}^{*}<R_{i}^{*}}\left[\left(\frac{q_{i^{\prime }}}{q_{i}}\times overlap_{ii^{\prime }}\times B_{i^{\prime }j}\left(y,t\right)\right)\right]\right) \end{array}$$

In this model, each species *i* in each patch *j* grows towards its equilibrium monoculture biomass (carrying capacity) in patch *j*, B*_ij_, minus effects caused by interspecific competition from all other species in the community, species *i’*, that have lower R*_i_ (i.e., superior resource competitors). Growth follows a linear differential equation (i.e., exponential decay towards equilibrium), with relative growth rate for species *i* in patch *j, RGR*_*ij*_. Competition‐induced biomass loss to superior competitors is a function of the relative tissue nitrogen concentration, *q*_*i*_, of each species (i.e., total nitrogen taken up per unit biomass of species *i’* vs that of species *i*), multiplied by the fraction of spatiotemporal overlap between the two species. Total soil N available in patch *j* modulates B*_ij_ and *RGR*_*ij*_ based on an empirically fitted regression model comparing growth and biomass in monocultures across a soil N gradient (Table 1). The model thus makes four assumptions: (1) each species has access to some maximum amount of soil N in each patch, determined by *q*_*i*_ B*_ij_; (2) access to that nitrogen can be pre‐empted by superior competitors; (3) differences in species' spatiotemporal niches can partially shield poorer competitors from pre‐emption by superior competitors; and (4) nitrogen is ‘recycled’, such that declines in the biomass of species *i* immediately frees up nitrogen for other species. If the inclusion of these assumptions improves out‐of‐sample model predictions (tests described below), then that improvement can be taken as evidence of their relevance in our system. Note that previous studies have demonstrated the preeminence soil N competition in Cedar Creek grasslands (Fargione and Tilman 2005; Clark et al. 2018).

To simulate multi‐patch dynamics, we link the arrival and establishment submodels using a *lottery function* (Table 1), which incorporates stochasticity and impacts of priority and mass effects (respectively: species arrival order, which can affect the trajectory of community composition (Fukami et al. 2005); and source‐sink dynamics where surplus seed moves from a source to a sink (Leibold et al. 2004)). The function is not directly influenced by species competitive ability (R*) and thus can enable niche pre‐emption among seeds (but not among adults). In the lottery function, we randomly and uniformly sample from the pool of arriving seeds without replacement and allow new seeds to establish in the plot until all available soil N has been consumed. To do so, we assume that each unit of arriving seed biomass can contribute up to 1/*f*_*i*_ units of new adult biomass, provided there is sufficient soil N in the plot to sustain it. This conversion is roughly equivalent to assuming that species' populations are at equilibrium—that is, if *B*_*ij*_ units of adult biomass can produce *B*_*ij*_ *f*_*i*_ units of seed biomass, then *x*_*ij*_ units of arriving seed biomass should result in about *x*_*ij*_/*f*_*i*_ new units of adult biomass. Based on species' nitrogen content per unit biomass (*q*) and the adult biomass that each seed would produce, the lottery function considers how much nitrogen would be consumed by a seed of each species and subtracts that amount from the available soil N (which varies across species based on their R*). As more seeds colonise a patch, the “bucket” of soil N is drawn down, until no additional N is available to colonising species based on their R*, B* and/or seed rain. When holding species' R*, B* and *q* constant, the lottery function means that species that produce many seeds are more likely to colonise a plot (produce adult biomass) than species that produce few seeds.

### 2.2 Grassland Field Experiment

The experiment is summarised in Appendix S1, Tilman and Wedin (1991) and Wedin and Tilman (1993). Briefly, the experiment involved 43 seed sowing treatments, which manipulated species richness, species identity, species seedling ratio and species introduction sequence (Table S5). The five species used in the experiment are the most abundant grasses in old fields at Cedar Creek, though they vary in their successional status and response to nutrient addition: *Agrostis scabra* (native, C3 photosynthetic pathway) and *Elymus repens* (introduced, C3) are early successional species; *Poa pratensis* (introduced, C3) dominates mid‐successional fields; and *Schizachyrium scoparium* (native, C4 photosynthetic pathway) and *Andropogon gerardii* (native, C4) dominate older successional grasslands (Figure 1; for brevity, we refer to the species by their genera hereafter).

Seeds of the five grass species were sown in plots in May 1986, at a density to yield 600, 3000 or 6000 seedlings/m^2^. The total density of seedlings remained constant across the 1‐, 2‐, 3‐ and 5‐species mixtures, with just the ratio of seeds sown per species changing. Each treatment had between 1 and 4 replicates that were randomly assigned to 64 0.75 m × 0.75 m plots within each of 10 blocks. The blocks were separated by 1 m wide walkways, but the plots were contiguous within each block (see Appendix S1; Figure 1 for layout). The ten 3 m × 12 m blocks covered a gradient of total soil nitrogen (~100 to ~1200 mg/kg N) in a split‐plot design where each block differed in soil fertility. The entire experiment was fenced to exclude all mammalian herbivores. Plots were weeded for the first 6 years of the experiment, removing species that had not been intentionally sown in the target plots. We accounted for this in the model by only letting sown species colonise each plot (seed rain could still vary among species and over time based on species' dispersal kernels, abundances and locations in the multi‐patch matrix). Species aboveground biomass was sampled each year in each plot by clipping a strip of vegetation at peak biomass, then sorting, drying and weighing it.

### 2.3 Model Parameterisation

Models were parameterised using attribute data collected at Cedar Creek unless otherwise noted (Appendix S1, Tables S1 and S4). This included traits underlying species' dispersal kernels (i.e., height of seed release, seed terminal velocity; Figure 1, Table 1). Where needed, we used monoculture data from the target experiment (excluding block 5, which was fertilised), but no data from the experiment's polycultures were used. Apart from the monocultures, the input data were thus independent of the experimental data used to evaluate the model, enabling out‐of‐sample predictions for the 2‐, 3‐ and 5‐species mixtures. Model simulations were initialised based on total soil nitrogen measured in each plot and seed biomass of each species sown in each plot at the start of the experiment.

### 2.4 Model Performance

**FIGURE 2:**
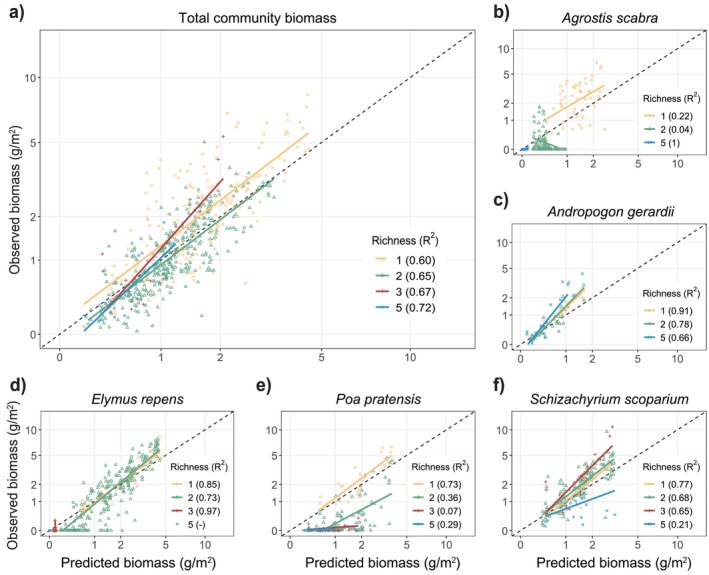
Observed versus predicted aboveground biomass (log g/m^2^), scaled by monoculture biomass, of five grass species in 1‐, 2‐, 3‐ and 5‐ species mixtures along a soil nitrogen gradient 6 years after sowing seeds for (a) all species mixtures, (b) *Agrostis scabra* , (c) *Andropogon gerardii* , (d) *Elymus repens* , (e) *Poa pratensis* and (f) *Schizachyrium scoparium*. Model predictions were based on the full multi‐mechanism niche model with all mechanisms and attributes included. For panels b to f, we divided observed and predicted biomass by monoculture biomass of each species (observed/B* vs predicted/B*), whereas for panel a, we summed across all five species (sum(observed)/sum(B*) vs sum(predicted)/sum(B*)). *R* ^2^ is shown for each level of species richness and gives an indication of the variance explained. Panels b to f only show scenarios that were included in the empirical experiment. Note log scale on both axes.

We assessed model performance by comparing species predicted and observed biomasses, each scaled by species monoculture biomass (B*), for each of our modelling scenarios and each experimental treatment at the end of year 6 (Figure 2). Year 6 was the last year of complete data following the experiment's original design. We did not in any way ‘tune’ or train the model to match predictions (Clark et al. 2018). As such, higher model complexity does not necessarily lead to better performance for out‐of‐sample predictions as would be expected for a statistical or phenomenological model. We ran 10 replicate simulations for each of the 2048 (2^11^) model scenarios and compared model fit when different combinations of the 11 attributes were switched off in a full factorial design (Figure 1, Appendix S1). This reveals the relative influence of each attribute on community composition (Gotelli et al. 2009). If critical to shaping composition, model performance would decline when a given attribute is turned off; if not influential, model performance would not change. We also specifically examined mechanism‐level effects by switching on/off the attributes representing each of the four mechanisms. We considered how many attributes of a mechanism were required for a mechanism to improve model fit.

To determine the relative importance of different attributes and mechanisms in the model, we identified the top 5% best biomass predictions (as indicated by the lowest root mean square error (RMSE)) for each plot for each level of model complexity, where model complexity is defined by total number of attributes or mechanisms included in a model (i.e., 1–11 attributes, 1–4 mechanisms). We then examined which attributes and mechanisms were switched on in the model runs that produced those predictions. We assessed model performance using *R* ^2^ and RMSE (Bennett et al. 2013). As RMSE scales with average biomass, we calculated it as:

$$\text{RMSE}=\frac{y_{ij}-{\hat{y}}_{ij}}{B_{ij}^{*}}$$

where *y*_*ij*_ is a species observed biomass in patch j, *ŷ*_*ij*_ is a species predicted biomass, and B*_ij_ is the species monoculture biomass. When not focusing on species‐specific errors, we averaged species RMSE values to get an average community‐level error per plot. Except where monocultures are explicitly shown, all results reported are based on polycultures only.

## 3 Results

The performance of our mechanistic model was high, accurately predicting aboveground biomass of each grass species 6 years after their seeds were sown in 43 experimental treatments along a gradient of soil N (Figure 2; Figure S1). Comparing predicted versus observed aboveground biomass, scaled by monoculture biomass, the overall *R* ^2^ value in multispecies plots was 0.65 when all model components were included (overall *R* ^2^ of 0.67 when including monocultures). Performance did vary among species and with plot species richness though (Figure 2).

**FIGURE 3:**
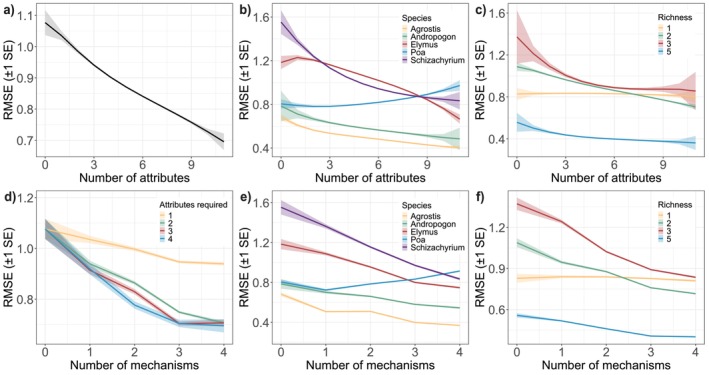
Model error when different numbers of attributes (upper panels) and mechanisms (lower panels) in the model are switched on. Panels show root mean square error (RMSE) of model predictions compared with observed biomass overall (a, d), for each sown species (b, e), and for each sown richness level (c, f). Error bars are larger at high numbers of attributes, as there are fewer combinations of attributes across which to average model performance. RMSE rather than *R* ^2^ is shown to avoid potential correlations between *R* ^2^ and number of replicates. RMSE is a relative measure, so comparisons are only valid within individual panels. Recall that the model was not ‘tuned’ to fit predictions to observations—as such, increases in model complexity need not reduce model error. Data from monocultures (which were used to parameterise models in some cases, Table S1) are excluded from results except where explicitly shown in panels c and f (i.e., richness = 1). In d, colour refers to the number of attributes within a mechanism that are required to ‘switch on’ a mechanism. In e and f, for a mechanism to be switched on, it must contain exactly *two* attributes per mechanism (results are comparable when 1, 3 or 4 attributes are included per mechanism, Figure S3).

**FIGURE 4:**
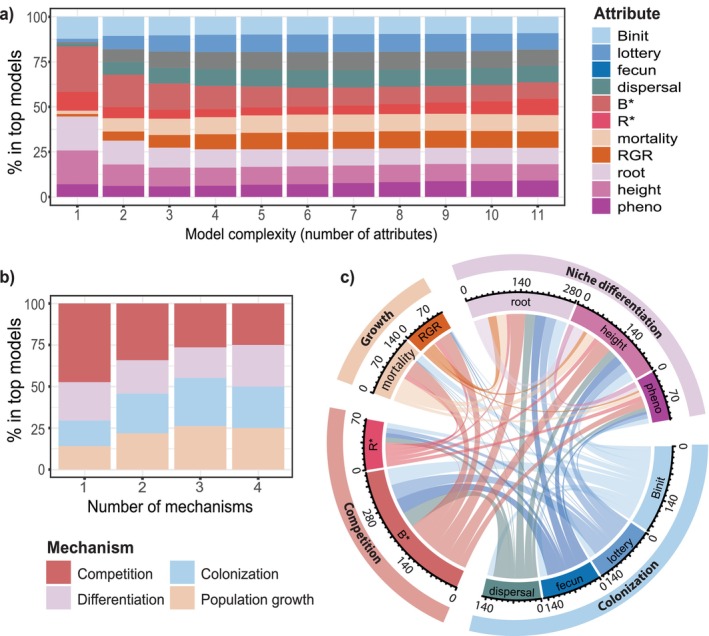
Frequency that attributes co‐occur in top‐performing models, assessed at the plot‐level, with (a) 1–11 attributes, (b) 1–4 mechanisms and (c) two attributes. Results show estimates of sown species and in plots with at least two sown species (i.e., polycultures only). Panels a and b show proportion that each attribute or mechanism appears in the top 5% best performing models for each level of model complexity based on RMSE. In b, for a mechanism to be considered ‘on’, it must contain exactly *two* attributes per mechanism (results are comparable when 1, 3 or 4 attributes are included per mechanism, Figure S9). In c, scales represent the number of times a pairwise combination occurs, with a total of 1,065 pairwise combinations (see Table S2 for details).

Overall, and across all species mixtures and all species except *Poa*, model performance increased with higher model complexity, indicated by declining RMSE as more attributes and mechanisms were switched on (Figure 3; Figures S3 and S4). Despite the superior performance of the full model, predictions from simpler models were still reasonable, especially if three of the four mechanisms (resource competition, colonisation, spatiotemporal niche differentiation, population growth rates) were represented by at least two attributes each (Figure 3d). There was variation in the relative importance of attributes and mechanisms in the best‐performing simple models (indicated by specific mechanisms and attributes being overrepresented in the top‐performing model variants when attributes were factorially excluded; Figure 4a,b; Figures S6–S11). For one‐ and two‐mechanism models, resource competition was somewhat more important than the other mechanisms (Figure 4b), and competition and colonisation most often featured together in the best two‐mechanism models (Figure S5). Once three mechanisms were used, the exact combination of mechanisms was less important though, and no single mechanism or attribute was essential—similar predictive performance could be achieved from numerous model configurations (Figure 4bc). This implies complementarity among mechanisms and interchangeability within them.

Pairs of attributes from different mechanisms typified the best two‐attribute models (Figure 4c, Table S2), further suggesting complementarity among the four mechanisms. Attributes representing competition and colonisation were especially overrepresented in the top‐performing two‐attribute models (namely B* with lottery, dispersal, *B_init_* or fecundity; Figure 4, Figure S5, Table S2). Collinearity was high among the 11 attributes (overall mean |*r*| = 0.51, Table S3) but was especially high among attributes from the same mechanism (mean |r| = 0.71, vs 0.46 among attributes representing different mechanisms). This implies that attributes from different mechanisms were more likely to capture unique information.

The attributes and mechanisms most often found in the best‐performing simple models varied by species and combinations of species. For example: B* was particularly ‘influential’ for *Elymus* and for the *Elymus‐Schizachyrium* pair (Figures S6i,j and S7c); R* was the most influential attribute for *Agrostis* at low model complexity (Figure S7a) and for the *Elymus‐Agrostis* pair (Figure S6a,b); root and height overlap were most influential for the *Elymus‐Poa* pair (Figure S6e,f); and all 11 attributes were equally influential for *Schizachyrium* (Figure S7e). Given life history differences in our five study species, it is not surprising that mechanisms and attributes involved in their co‐occurrence differed. However, there were no obvious relationships between species trait differences (Table S4) and the mechanisms and attributes that were most influential when modelling specific species pairs (Figures S6 and S12). Overall, species' carrying capacity in monoculture (B*) most often featured in the best‐performing single‐attribute models, followed by root and height overlap (Figure 4a).

## 4 Discussion

Our multi‐mechanism model accurately predicted out‐of‐sample aboveground biomass of five co‐occurring grass species along a gradient of soil nitrogen 6 years after their seeds were sown (Figure 2, Figure S1). By factorially switching on and off four mechanisms and 11 attributes that underpinned the model, and comparing predicted and observed biomass, we assessed model performance and determined the relative influence of different community assembly mechanisms.

The most striking findings of our study were: (i) the high out‐of‐sample predictive performance of the model (*R* ^2^ > 0.6, Figure 2a); (ii) the marked improvement in model predictions when including at least three of the four mechanisms; and (iii) the apparent interchangeability among the four mechanisms and their 11 underlying attributes in alternative variants of the model. The most parsimonious models consisted of six attributes shared equally among three of the four mechanisms (soil resource competition, dispersal and colonisation, spatiotemporal niche differentiation, population growth rates; Figure 3d). Provided enough mechanisms were represented, exactly which attributes and mechanisms were included rarely mattered: the model could still predict assemblage composition well. These findings are highly significant as they suggest that, because of complementarity among mechanisms and interspecific trait tradeoffs,—and provided enough core mechanisms are captured—we can accurately forecast community composition and in a flexible way.

Numerous models with different underlying mechanisms have been proposed to explain plant community dynamics (Tilman 2004; Vellend 2016; Leibold and Chase 2018). It has been unclear when and where each of these mechanisms matters most. In our case study, we found that different combinations of measured plant attributes and mechanisms seem to have similar predictive power in a multi‐mechanism model. This suggests that somewhat different multi‐mechanism models might perform similarly well when making anticipatory predictions (Mouquet et al. 2015). This is promising as it implies that exact model specification can be flexible, using mechanistic data that is most readily available for a given system. Given the low availability of some of our input data (e.g., competitive ability—R*), such flexibility could alleviate the need for comprehensive data collection prior to the development of useful models. However, because such models may not necessarily reflect the most important mechanisms in a system, their value for explanatory prediction (i.e., ecological and evolutionary understanding) may be limited; it may not be possible to use the predictive performance of a mechanistic model to indicate which mechanisms matter most. This in turn suggests that research that identifies specific mechanisms as being especially important for particular systems (e.g., competition for soil N in Cedar Creek grasslands, USA (Tilman 1982), plant–soil feedbacks in Jena grasslands, Germany (Weisser et al. 2017), Janzen‐Connell effects in Barro Colorado Island's tropical forests, Panama (Wright 2002)) might actually not be so definitive after all—perhaps other mechanisms would do just as well in explaining local community dynamics? And perhaps multiple mechanisms would do far better? These are controversial ideas that would need thorough testing.

### 4.1 Relative Importance of Ecological Mechanisms and Attributes

Each of the model's four mechanisms and 11 attributes helped to improve model accuracy and performance, implying that they each relate to the composition of multispecies grass mixtures at Cedar Creek. This finding is ecologically meaningful: because we tested out‐of‐sample fits, and because our models comprised a mix of non‐linear functions based on a priori biological expectations (i.e., they were not tuned to observational data and were not statistical models like pattern‐fitting regressions), there is no reason to expect that more complex models would make better predictions, as predictions of *Poa* illustrate (Figure 3, Figure S2–S4). The poor predictions of *Poa* biomass were unsurprising given field observations. *Poa* tends to fail in monoculture, making it hard to accurately quantify the traits that were used to parameterise the model.

The species‐ and richness‐level trends provide an explanation for why it was best, overall, to include a diversity of mechanisms in the model and why different combinations of mechanisms yielded similarly strong predictions (Figures S2, S3, S5–S7, S9 and S10). The relative importance of attributes and mechanisms differed among species and species combinations (Figures S6–S8 and S10–S12), suggesting that different species rely on different mechanisms for co‐occurrence and thus each mechanism captured some outcomes well and others poorly. Such inferences are consistent with theory, which posits that species coexistence and high biodiversity are enabled by myriad processes and life history or trait tradeoffs (Tilman 2004; Harpole et al. 2016). For instance, the observed overrepresentation of competition and colonisation in the top‐performing two‐attribute and two‐mechanism models is reminiscent of theory that links these processes via an interspecific tradeoff (Figure 4c, Figure S5a, Table S2; Tilman 1994).

Although model attributes were interchangeable, attributes related to resource competition and spatiotemporal niche differentiation most often featured in the best‐performing simple models (≤ 4 attributes, Figure 4a, Figure S6). It is likely that carrying capacity (B*) featured most overall because the model architecture specifies that it sets each species' maximal abundance and thus it has the greatest potential to modify species biomass. Results indicated that species' competitive ability for soil nitrogen (R*, Table 1) was less influential than B*. However, R* was still overrepresented in the best simple models (Figure 4a), emphasising the ecological (as well as methodological) importance of soil N competition in our study system relative to other mechanisms examined. Spatiotemporal niche differentiation effectively enables species to decrease the impact of resource competition (Wandrag et al. 2019; Levine et al. 2022), including competition for soil N. The influence of spatiotemporal niche differentiation in our model further underscores the likely importance of resource competition in these multispecies mixtures, consistent with prior findings (Wedin and Tilman 1993; Tilman 1994).

### 4.2 Model Novelty and Utility

Unlike most community assembly models, our model explicitly considered multiple mechanisms at once, was spatially and temporally explicit, and was evaluated with empirical out‐of‐sample data and not tuned (Blonder et al. 2024). Predictions of most mechanistic models are not quantitatively evaluated against observational data (Tilman 2004; Lerch et al. 2023), including at Cedar Creek (Tilman 1994; Dybzinski and Tilman 2007; Farrior et al. 2013), and direct comparisons between models are impossible when case studies and performance tests differ (e.g., Levine et al. 2022). However, our model performed well relative to comparable mechanistic models. Whereas our model had an overall *R* ^2^ of 0.65 in multispecies plots (Figure 2), Chalmandrier et al. (2021)'s model based on plant competition and temperature‐dependent growth, applied in the French Alps, returned a pseudo‐*R* ^2^ of 0.18 (this increased to 0.52 when tuned using a transfer function). Also working in Cedar Creek grasslands, Clark et al. (2018)'s model based on competition for soil N returned an *R* ^2^ of 0.52 when the model was parameterised using ‘raw’ trait data like we do here.

By examining all possible levels of model complexity in our study, we found that multi‐mechanism models were far better at predicting community composition than ‘traditional’ one‐ or two‐mechanism models (Figure 3). Indeed, we found that at least three of the four mechanisms we examined were required to make strong predictions of multispecies community assembly. Perhaps as importantly, we found that as few as six such attributes, shared across three mechanisms, could reasonably approximate the predictions of more complete models. The performance and flexibility of our model hold promise for more complicated applications and scenario modelling (Box 1). These include identifying mechanisms that may be important for species coexistence and diversity, extrapolating to novel communities and conditions and informing decision making under global environmental change.

BOX 1 **Can This Model Be Applied to Other Study Systems?**

Our study is unusual in that we applied and tested our multi‐mechanism niche model using an experiment of five grasses and in a system that has been extensively studied. (Cedar Creek has been an ecological research site since 1942 and became part of the US National Science Foundation Long Term Ecological Research Network in 1982). As such, in our study, we felt confident to focus on competition for soil N rather than other resources (Wedin and Tilman 1993; Tilman 1994; Farrior et al. 2013) and there was suitable data available to parameterise the models (Clark et al. 2018; Sullivan et al. 2018; Catford et al. 2019). The wealth of knowledge and understanding of Cedar Creek grasslands may have facilitated our high model performance. Most models would be applied to more diverse natural systems where knowledge and data is less available—so, how could our model be adjusted to suit them?

The structure of our model should be broadly applicable, reflecting the widespread importance of the four key mechanisms—resource competition, dispersal and colonisation, spatiotemporal niche differentiation, population growth rates—for plant communities (Vellend 2016). However, some adjustments would likely be necessary to reflect the ecology of specific systems and data availability. Resource competition should reflect the limiting resource/s of a system, so R* (competitive ability for soil N) may need to be replaced by another attribute (e.g. L* in light‐limited systems (Dybzinski and Tilman 2007), or other similar metrics (Levine, An, et al. 2025)). Although our study suggests there is redundancy among attributes, there may be a need to incorporate additional attributes to capture, for example, competition for multiple resources (Harpole et al. 2016) or dispersal through time (Wisnoski and Shoemaker 2022). It would be useful to assess model sensitivity to input data, including traits measured in monoculture vs polyculture. When parameterisation data are limited, ‘soft’(er) functional traits could serve as surrogates for ‘hard’(er) traits (Belluau and Shipley 2018). For example, dispersal kernels could be characterised with growth form, seed mass and dispersal mode, thus avoiding the need for terminal velocity (Tamme et al. 2013), and belowground biomass allocation could be used in place of R* (Tilman 1994). Extending beyond species‐level, species could be categorised into groups (or ‘syndromes’ (Agrawal 2020)), enabling estimates of group‐based trends (Gregory et al. 2025). When specific mechanisms are poorly understood (e.g. competition in some systems), there is also the option to incorporate correlative elements into the model (Briscoe et al. 2019).

Mechanistic niche models hold great promise for modelling a broad range of species and systems, including those at non‐equilibrium (Fenollosa et al. 2025). We believe that the structure and configuration of our multi‐mechanism niche model should make it readily adaptable to other situations.

## 5 Conclusion

Our study points to the multidimensional nature of community assembly. It demonstrates that plant attributes that directly relate to mechanisms of species interaction and community assembly can accurately predict species abundances in a multispecies system, and that a suite of such mechanisms can create high‐performing ecological models that are tractable and realistic. While a complex model is better for explanatory prediction, our study suggests that simpler, more parsimonious models can be useful for anticipatory prediction. Our study also highlights that alternative combinations of ecological mechanisms and attributes can underpin robust predictive models if enough core mechanisms are adequately captured. Thus, rather than trying to identify a unique set of 'best' attributes and ‘best’ mechanisms for predicting community assembly, modellers and managers may be better served by comparing predictions from a wide range of multi‐mechanism models. It, of course, remains to be seen how broadly these results hold for other locations, species and model mechanisms—we strongly encourage others to test similar, multi‐mechanism community assembly models in other systems.

## Author Contributions

Jane A. Catford conceived the idea and led initial model development with input from Cindy E. Hauser, Nicola T. Munro and Brendan A. Wintle. Adam T. Clark, Laura J. Graham and Jane A. Catford revised the model framework. Adam T. Clark and Laura J. Graham implemented the model and wrote the code. Adam T. Clark and Laura J. Graham parameterised the model and ran simulations, with input from Jane A. Catford, Harry E.R. Shepherd and John G. Donohue. David Tilman designed and established the empirical experiment. Adam T. Clark, Jane A. Catford, Nicola T. Munro and David Tilman collected and compiled data used to parameterise and test the model. Laura J. Graham, Adam T. Clark and Harry E.R. Shepherd analysed model results with input from Jane A. Catford. Jane A. Catford, Harry E.R. Shepherd, Laura J. Graham and Adam T. Clark created figures. Jane A. Catford wrote the first draft of the paper with input from Adam T. Clark and Harry E.R. Shepherd. All authors contributed to revisions.

## Funding

This project has received funding from the European Research Council (ERC) under the European Union’s Horizon 2020 research and innovation programme (Grant [101002987]), the Australian Research Council (ARC, DE120102221), the ARC Centre of Excellence for Environmental Decisions and the US National Science Foundation Long‐Term Ecological Research (LTER) Program (including DEB‐2425352, DEB‐0620652, DEB‐1234162 and DEB‐1831944). Funding for the project comes “from the European Research Council (ERC) under the European Union’s Horizon 2020 research and innovation programme (grant agreement No. [101002987]).” Cedar Creek Ecosystem Science Reserve, the University of Minnesota and King’s College London provided further support.

## Supporting information


**Data S1:** ele70358‐sup‐0001‐supinfo.pdf.

## Data Availability

Empirical data for the grassland experiment (e026) are available here: https://cedarcreek.umn.edu/research/data. All data and code needed to replicate the analyses are available in github archives (https://github.com/laurajanegraham/simulateCoexistence and https://github.com/hshepherdeco/Predicting_e026_coexistence) and as a permanent archived version on Zenodo (https://doi.org/10.5281/zenodo.15295835).
