# Supplementary material for: Multiple Mechanisms Required to Predict Grass Community Composition
Source: Ecol Lett. 2026 Apr 12;29(4):e70358. doi: 10.1111/ele.70358 (PMC13070581; doi:10.1111/ele.70358)
Supplement: Supplementary file 1 — Data S1: ele70358‐sup‐0001‐supinfo.pdf. [file ELE-29-0-s001.pdf]

Supporting Information for

Catford, J.A., Graham, L.J., Shepherd, H.E.R., Hauser, C.E., Munro, N., Wintle, B.A., Donohue, J., Tilman, D. & Clark, A.T. (2026) Multiple mechanisms required to predict grass community composition. *Ecology Letters*.

Corresponding authors: Jane A. Catford (jane.catford@anu.edu.au)
Adam T. Clark (adam.clark@uni-graz.at)

This PDF file includes:

Appendix S1
Figures S1 to S14
Tables S1 to S6
SI References

Appendix S1: Additional methodological details about grassland experiment, model parameterization, model performance and model attribute switches

Grassland experiment

The grassland plant community field experiment was conducted at Cedar Creek Ecosystem Science Reserve, Minnesota, USA. For full study details and key results of the seeding experiment, see (Wedin & Tilman 1993). Briefly, the experiment consisted of eleven 3 m x 12 m blocks, with blocks separated with 1 m-wide walkways (Appendix S1 Fig. 1, below), each of which was randomly assigned to a different soil mixture treatment that varied in ratio of topsoil and sand. This created a gradient in total soil nitrogen that varied from ~100 mg/kg N (100% sand) to ~1200 mg/kg N (100% topsoil); we excluded block 5 from our analyses, which was fertilised to raise soil N further, and block 11, which was burned. B* values used in the model were adjusted to account for the soil N gradient based on log-transformed linear regressions of species monoculture biomass vs soil nitrogen concentration (described in Table S1; see code for details). All blocks had a soil pH of 7.2, received additional micronutrients to ensure that N was the only nutrient limiting plant growth, and were irrigated during the growing season. At the start of the experiment in spring 1986, each block was divided into 64 0.75 m x 0.75 m experimental plots by driving galvanised sheet metal 0.23 m into the soil. The entire experiment was fenced to a height of 2 m and to a depth of 1.3 m to exclude all mammalian herbivores. The experiment included 43 seed sowing treatments, which manipulated species richness, species identity, species seedling ratio and species introduction sequence (details in Table S5). Each treatment had between 1 and 4 replicates per block. The 43 treatments were randomly assigned to the 64 plots within a soil mixture block, with each block being independently randomised.

Seeds of the five grass species were planted in plots in late May 1986, at a density to yield 600, 3000 or 6000 seedlings/m^2. The total density of seedlings remained constant across the 1-, 2-, 3 and 5- species mixtures, with just the ratio of seeds sown per species changing. There were two replicates per block for both the 5-species mixture and the (single) 3-species mixture, the latter of which consisted of *Poa, Elymus* and *Schizachyrium*. All species were sown at the same ratio in the 3- and 5-species mixtures (i.e. 1:1:1 and 1:1:1:1:1). Six pairs of species were used in the 2-species treatments (pairs shown in Fig. S4), each with one replicate per block except for the *Elymus-Agrostis* treatments, which had three replicates per block. Three seedling ratios were

used for each of the six 2-species mixtures (1:4, 1:1, 4:1), resulting in 18 2-species treatments. There were between two and seven replicate monocultures for each of the five species (Table S5). Plots were weeded throughout each growing season for the first six years of the experiment, removing species that had not been intentionally sown in the target plots. We accounted for this in the model by only letting sown species colonize each plot. Species aboveground biomass was sampled each year in each plot by clipping a designated strip of vegetation at peak biomass, then sorting the dry biomass to species, weighing and converting into g/m^2. More details in (Tilman & Wedin 1991b, a; Wedin & Tilman 1993) and https://cedarcreek.umn.edu/research/experiments/e026.

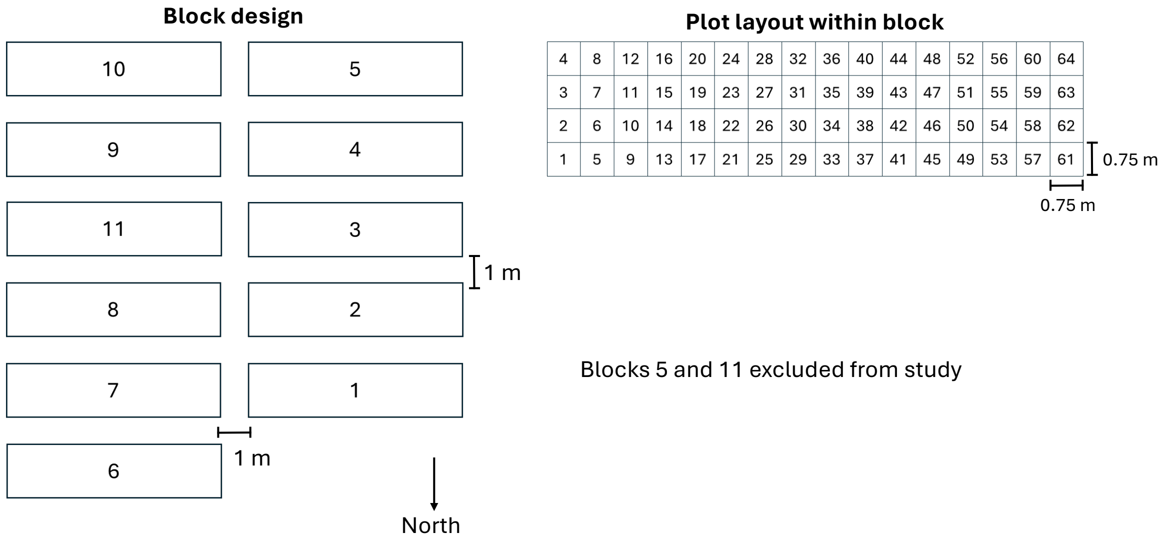

Appendix S1 Figure 1: Layout of experiment E26 showing blocks and plots. Block 5 and block 11 were not included in our study.

Input data and model parameterization

Wherever possible and unless specified, we calculated mean trait values based on observations from monoculture, as summarised in Table S1 and the “data-raw” folder in the archive and processed by the “DATASET.R” script. In rare cases where we did not have species-level trait data for all five study species, we used a wider trait dataset of 105 species to parameterize a multivariate normal distribution based on the relationship observed across all 5 species and the 12 traits in our dataset. We then calculated the conditional mean estimates for each missing species–trait combination, based on the

conditional distribution and the available trait data for each species. This approach effectively assumes that species traits fall along a high-dimensional trade-off surface, as demonstrated for grassland plant species at Cedar Creek by Clark *et al.* (Clark *et al.* 2018) and for plant species in general by Wright *et al.* (2004) and Reich (2014). Trait values, and their correlations, for the five study species are shown in Tables S4 and S6.

Model performance and relative importance of model attributes

For each of the 43 seed sowing treatments and soil N levels, we compared observed and predicted aboveground biomass of the sown grass species in plots at the end of year 6 (Fig. 2). We concentrate on results from year 6, which was the last year of complete data following the experiment's original design. We did not "tune" the model to match predictions, so higher model complexity does not necessarily lead to better performance for out-of-sample predictions. Note that simulations of the underlying differential equations governing within-growing season vegetation dynamics (i.e. of $B_{ij}(y,t)$) were conducted using log-transformed per-capita growth rates to avoid numerical instability, and then back-transformed after the simulation was complete to extract biomass estimates. This approach is possible because, via the chain rule, d(log(x))/dt = dx/dt/x. This approach allows the ODE solver to take slightly longer steps without risking finite computational errors leading to negative abundances, and thus causes the ODE solver to run much faster and more stably, but does not otherwise influence predicted dynamical trajectories.

As well as comparing observed biomass with the biomass predicted by our full model, where all 11 attributes were switched on (Figs 2 & S1), we compare model fit when different combinations of the 11 attributes were switched off in a full factorial design (Figs 3, 4, S2-S14, switches described below). This reveals the relative importance of each of the attributes to community assembly. If critical to community assembly, model performance would decline when a given attribute is turned off; if not influential, model performance would not change. We also specifically examined mechanism-level effects, by switching on/off the attributes representing each of the four mechanisms. We considered all possible criteria for a mechanism to be considered "on"; i.e. when one, two, three or all four attributes for a mechanism were switched on.

We ran 10 replicate simulations for each of the 2048 (2^{11}) model scenarios with attributes factorially switched on/off. This produced a total of 10 biomass predictions for

each scenario, species and year for each of the 576 experimental plots. By year 6, the year on which are results are based, the standard deviation from replicate simulations was 5 x 10^{-6} g m^{-2} of biomass, which equates to an average deviation of 0.07% (i.e. deviation relative to estimated biomass), so 10 replicates was sufficient to capture the trends. To determine the relative importance of different attributes in the model, we identified the top 5% best biomass predictions (as indicated by the lowest RMSE, described below) for each plot for each level of model complexity. We then examined which attributes were switched on in the 2043 model runs that produced those predictions. All possible combinations of attributes were considered for each level of model complexity (i.e. same number of attributes switched on/off but with attribute identity varying factorially). We did this overall and for each species, each richness level and each pairwise combination of species for 2-species plots. We also did this at the mechanism-level, such that we could identify which mechanisms enabled the most accurate predictions for each level of model complexity (i.e. 1-4 mechanisms).

We assessed model performance by comparing species' predicted and observed biomasses, each scaled by species monoculture biomass (B*), for each of our modelling scenarios and each of the experimental treatments. We assessed model performance using R^2 and root mean square error (RMSE), which is the standard deviation of the residuals. RMSE accounts for differences in species' biomass values but does not indicate whether the model over- or under-predicts species biomass. As RMSE scales with average biomass, we divided observed and predicted biomass values by species monoculture biomass (B*), such that:

Species RMSE = $(|y_i - \hat{y}_i|) / B^*_i$

where y_i is a species observed biomass, $\hat{y}_i$ is a species predicted biomass, and B^*_i is a species monoculture biomass. Since we had one observed biomass estimate per species per plot, then this is equivalent to a species predicted RMSE. For each plot, when not focusing on species-specific errors, we averaged species RMSE values to get an average error per plot.

Model attribute switches

Grouped into their four mechanisms and in addition to information provided in Table 1 in main text, the 11 attributes were switched off as follows:

Mechanism 1: Soil resource competition

i) *Nitrogen competition (R*):* setting "switch_off_rstar" to TRUE sets the competitive hierarchy for nitrogen to be equal across all species, implemented by setting intraspecific interaction strength to equal interspecific interaction strength.

ii) *Carrying capacity (B*):* setting the "switch_off_bstar" switch to TRUE sets the carrying capacity to be equal across all species.

Mechanism 2: Dispersal and colonization

i) *Lottery function:* To germinate, seeds that are produced by each species compete via a lottery function for unused resources in each plot. When holding species' resource needs (q) constant, species that produce many seeds are more likely to colonize a plot (produce adult biomass) than species that produce few seeds following random uniform draws from the full seed pool. Setting "switch_off_lot" to TRUE turns off the lottery function (random draws), such that unused resources are instead divided deterministically among species, in proportion to their seed production. When there is high resource availability relative to species resource needs (q), the random and deterministic draws would produce similar results. When resources are limited, the lottery function can be more influential.

ii) *Dispersal*: Seeds can disperse among plots following the dispersal kernel described in the arrival submodel. Setting the "switch_off_dispersal" to TRUE turns off between-plot dispersal, such that seeds must stay in the same plot in which they were produced (effectively isolating plots from one another).

iii) *Initial biomass:* Species initial biomass is set based on the experimental seeding rates for each plot. Setting the "switch_off_b_init" to TRUE sets initial abundance to be equal across species regardless of initial seeding rate.

iv) *Fecundity*: Setting "switch_off_rep_local_diff" to TRUE sets reproduction rate to be equal across all species (such that X grams of adult plant produces X grams of viable seed for the next generation for all species).

Mechanism 3: Spatiotemporal niche differentiation

Spatiotemporal niche differentiation reduces competitive impacts among species, proportional to species' belowground, aboveground and phenological overlap. For example, if species A roots to 50 cm and species B roots to 100 cm, the competitive impact of species B on A would be weighted at 100% because 100% of species A roots would be affected by species B roots, whereas the impact of A on B would be reduced by 50%. Similarly, if species C is active from June to August and species D is active from May to September, impacts of C on D would be weighted at 60%, whereas impacts of D on C would be weighted at 100%.

v) *Root overlap (belowground spatial partitioning):* setting "switch_off_root" to TRUE turns off differentiation in rooting depth among species; when FALSE, species can differ in their rooting depths, and competition is scaled relative to their vertical root overlap.

vi) *Height overlap (aboveground spatial partitioning):* setting "switch_off_height" to TRUE turns off differentiation in plant height among species; when FALSE, species can differ in their heights, and competition is scaled relative to their height overlap.

vii) *Phenology overlap (temporal partitioning):* setting "switch_off_temp" to TRUE turns off interspecific differentiation in phenology; when FALSE, species can differ in their phenology, and competition is scaled relative to their phenological overlap.

Mechanism 4: Population growth rates

viii) *Mortality*: Setting "switch_off_mor_diff" to TRUE turns off differences in mortality rates among species; when FALSE, mortality rates vary across species.

ix) *Relative growth rates:* Setting "switch_off_rgr" to TRUE sets all growth rates to be equal across species, based on the average growth rate across all species.

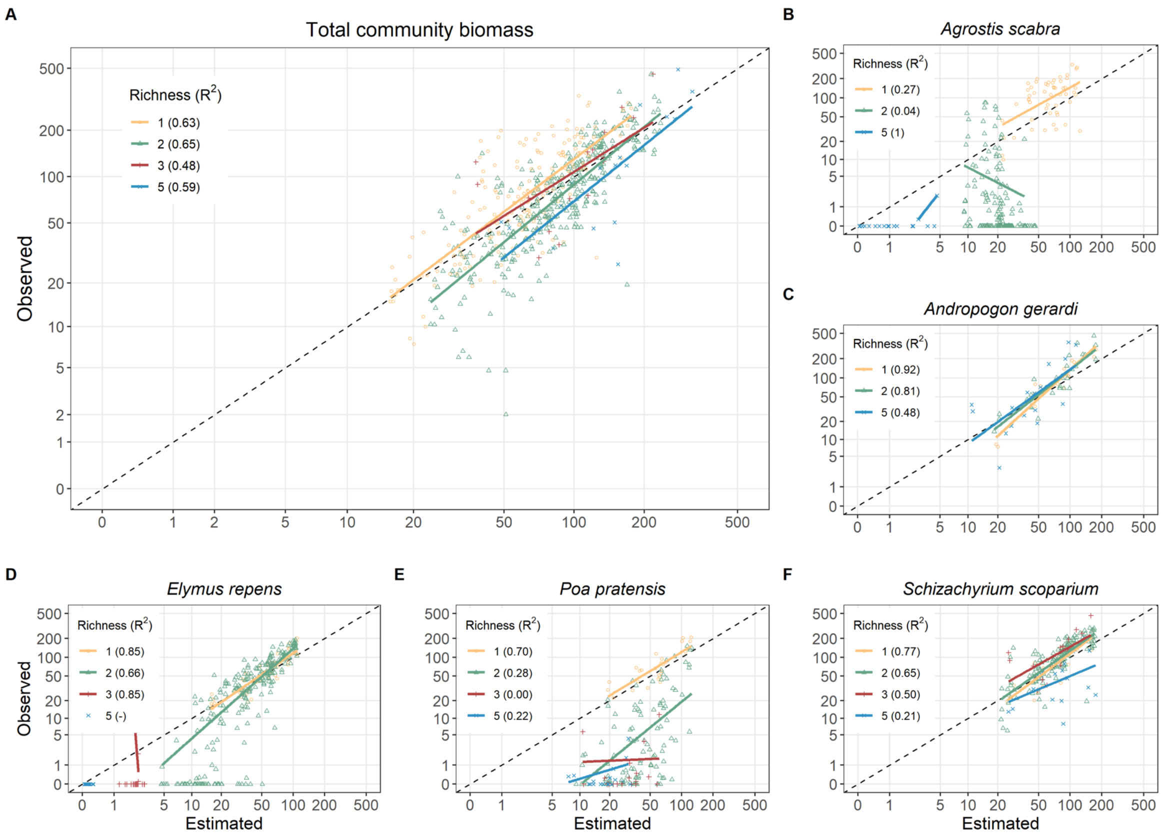

Figure S1: Observed versus estimated aboveground biomass (log g/m^2) of five grass species in 1-, 2-, 3 and 5- species mixtures along a soil nitrogen gradient six years after sowing seeds for a) the full community, b) *Agrostis scabra*, c) *Andropogon gerardii*, d) *Elymus repens*, e) *Poa pratensis*, and f) *Schizachyrium scoparium*. Model estimates were based on the full mechanistic niche model with all mechanisms and attributes included. R^2 is shown for each level of species richness. b-f only show scenarios that were included in the empirical experiment. Note log scale on both axes. Panels compared predicted vs observed when values are NOT scaled by monoculture biomass (cf Fig. 2 in main text).

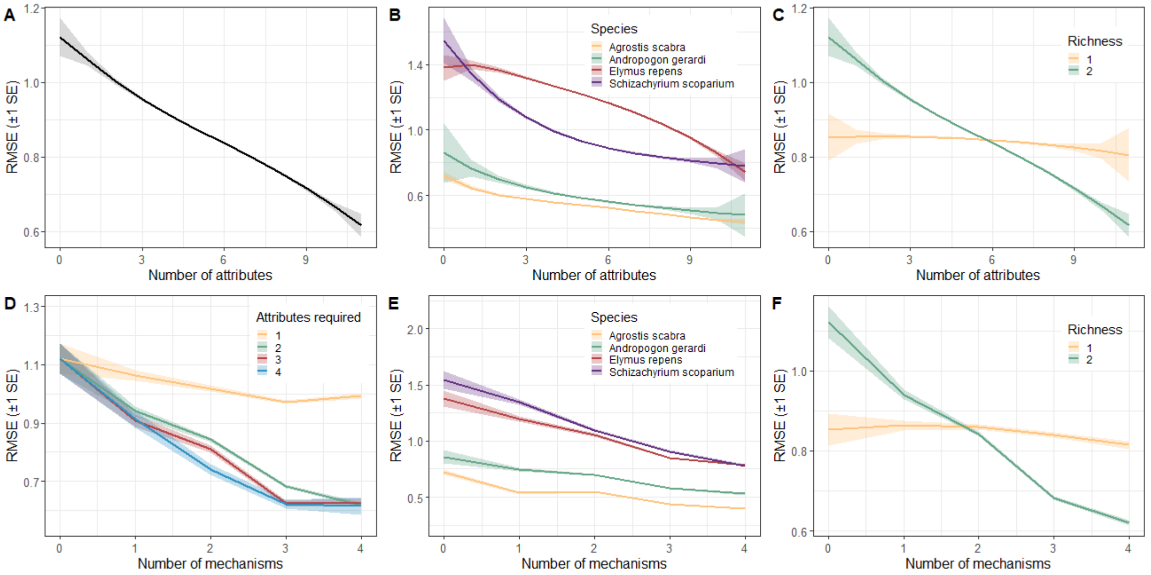

Figure S2: Model error when different numbers of attributes (upper panels) and mechanisms (lower panels) are included in the model, excluding plots where *Poa pratensis* was sown. As *Poa* is sown in all plots with 3 species, panels a, b, c & d only include estimates from 2+ species plots (single species plots are removed). Panels show root mean square error of model estimates compared with observed biomass overall (a, d), for each sown species (b, e), and for each sown richness level (c, f). Error bars are greater at high and low values as there are less replicates. RMSE is a relative measure, so comparisons are only valid across the same dataset. Data from monocultures are excluded from results except in panels c & f. In d, colour refers to the number of attributes within a mechanism category required to turn on a mechanism. Where a mechanism contains less than the specified number of attributes (such as growth, which only contains 2 attributes), then it contains all of the attributes. In lower panels, for a mechanism to be switched on, it must contain two attributes for each mechanism.

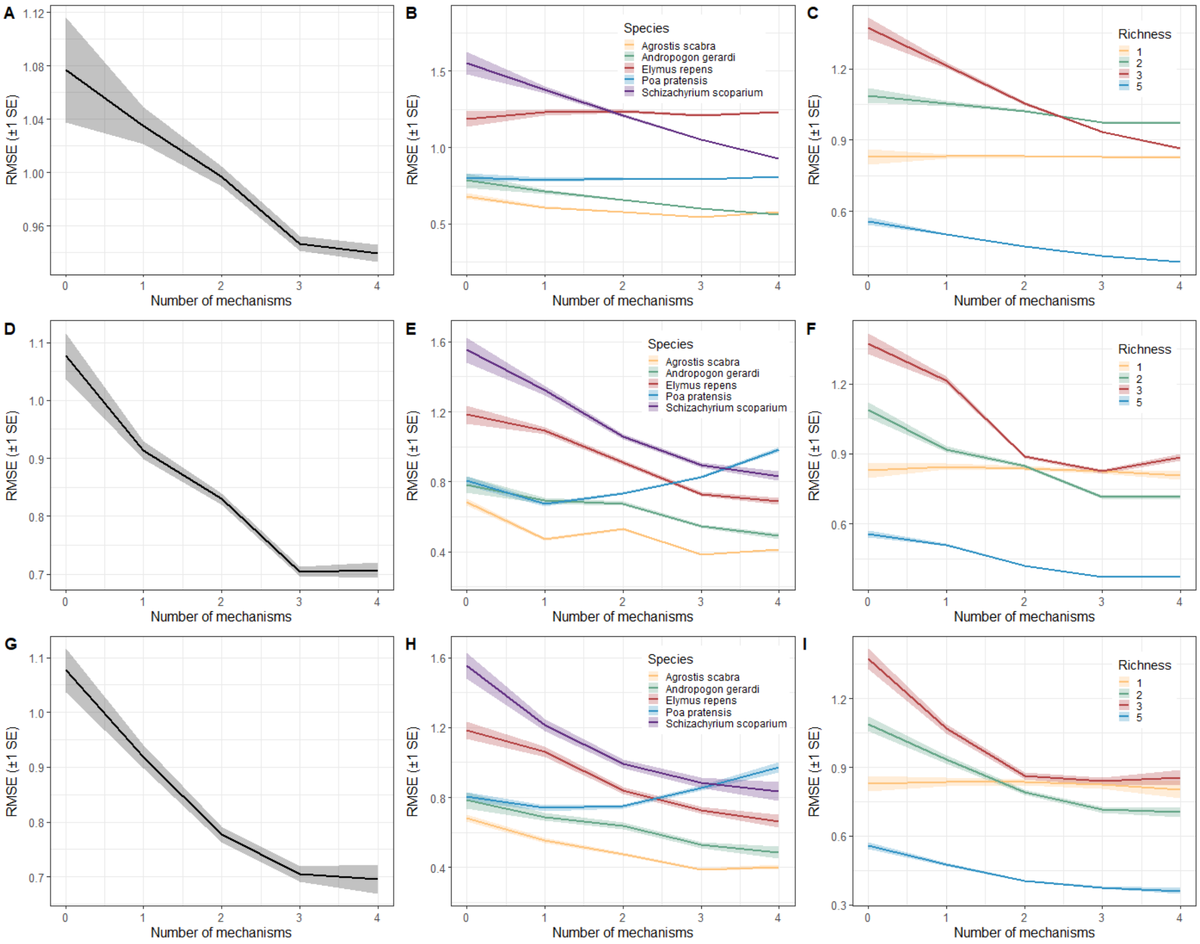

Figure S3: Model error with different number of mechanisms included in the model. For a mechanism to be included, it must contain one (upper panels), three (middle panels) or four attributes of each mechanism (lower panels). Where a mechanism contains less than the specified number of attributes (such as growth, which only contains 2 attributes), then it contains all of the attributes.

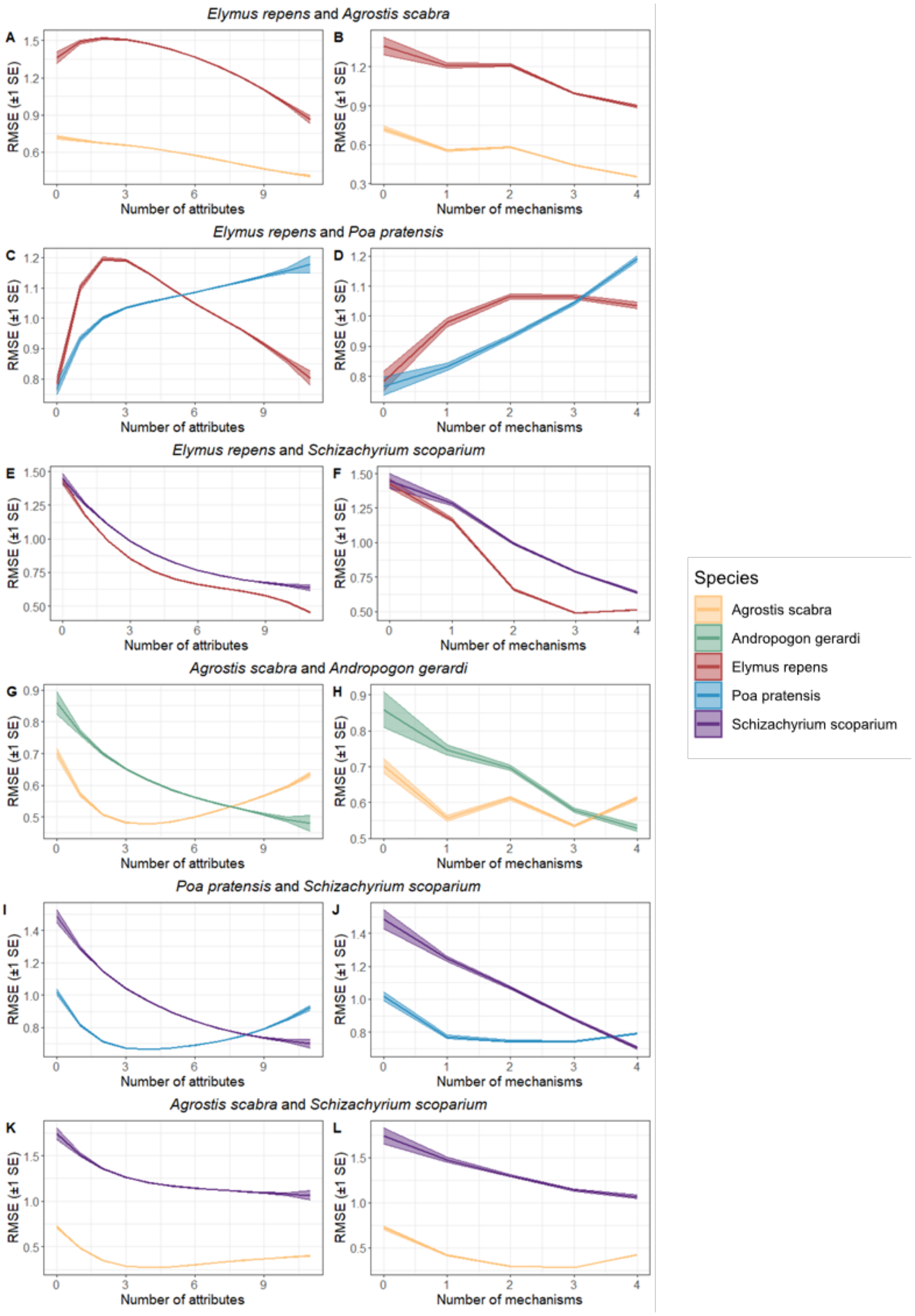

Figure S4: Species error with increasing model complexity for two-species plots, split with species ID with increasing numbers of attributes (left panels) and mechanisms (right panels). For a mechanism to be considered included in the model, it must contain <u>*two*</u> attributes from a mechanism.

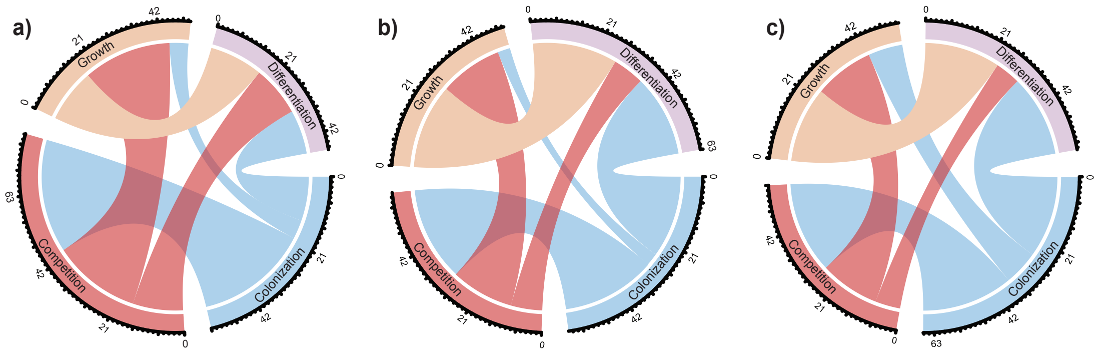

Figure S5: Frequency in which different mechanisms co-occur in models within the top 5% of simulations when two mechanisms are included. For a mechanism to be included, it requires a) two attributes, b) three attributes or c) four attributes from a mechanism. The scale in each panel represents the number of times two mechanism combinations occur, with a total of 117 pairwise combinations in each panel.

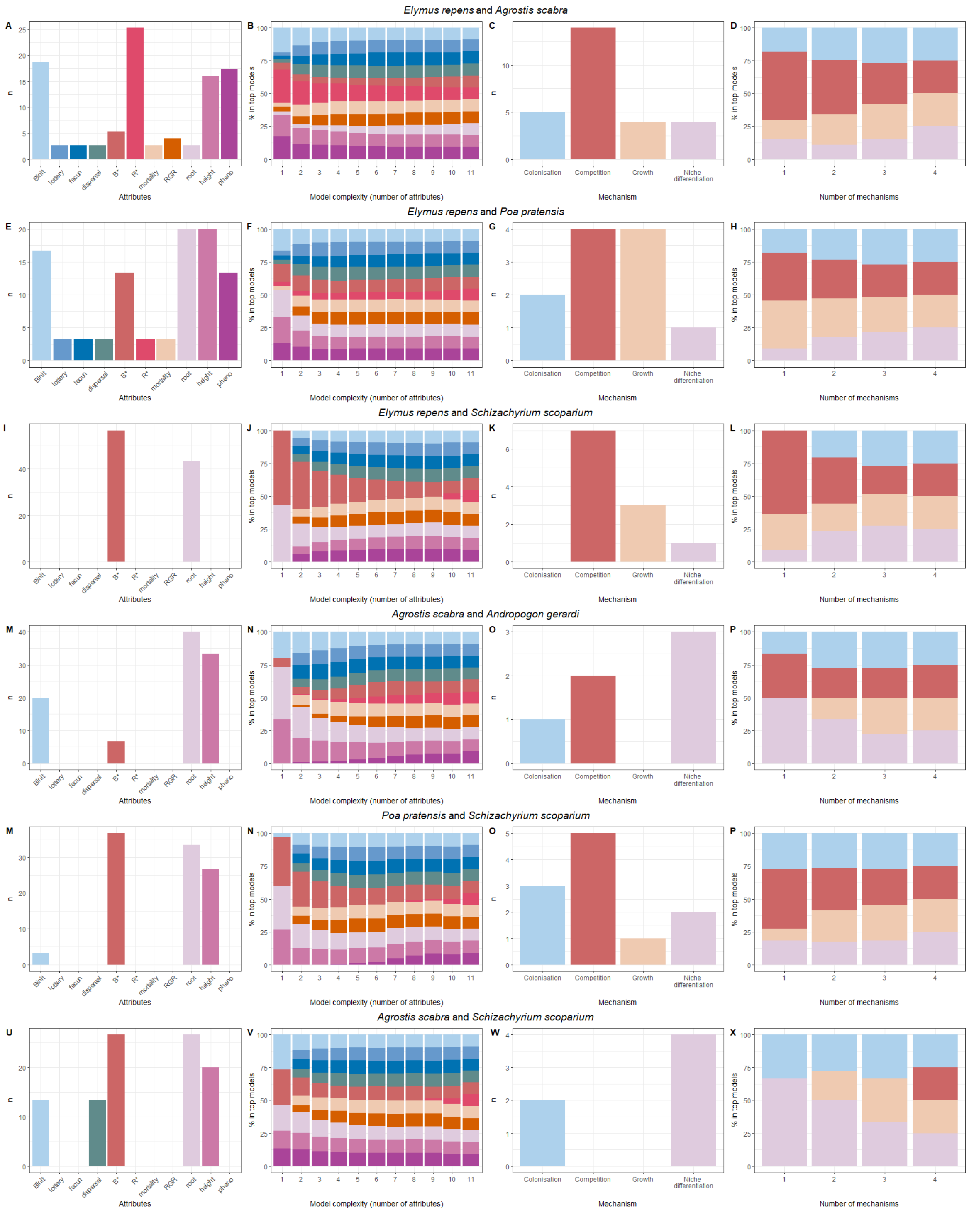

Figure S6: Frequency with which each mechanism occurs within the top 5% of best performing models with a single switch turned on (first and third columns) and percentage that each switch appears in the top 5% of best performing models for each number of switches (second and fourth columns) for attributes (left panels) and mechanisms (right panels) for each 2-species mixture. Results show estimates of sown species and in plots with only two sown species. For a mechanism to be switched on, it must contain *two* attributes per mechanism.

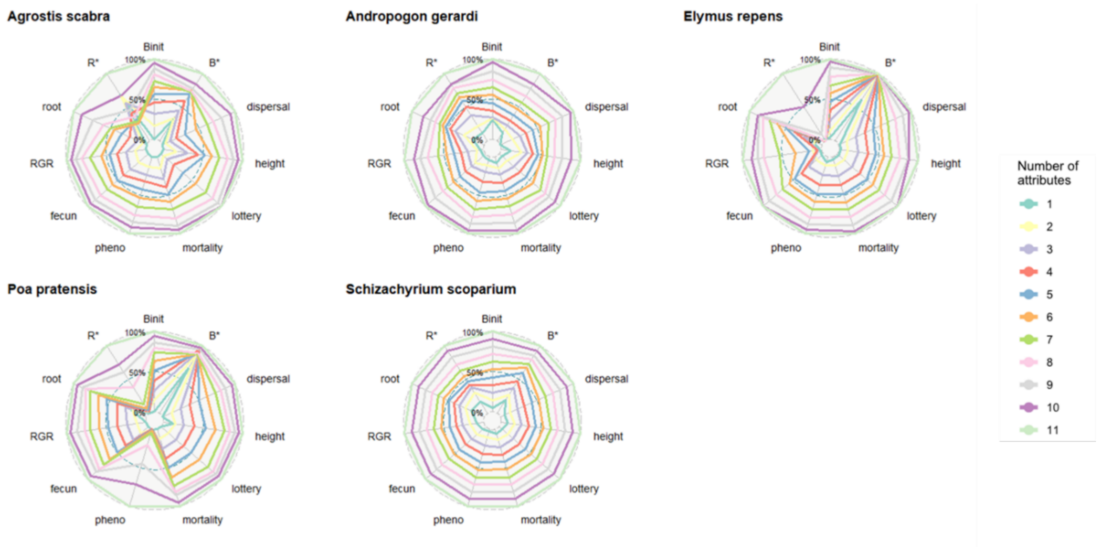

Figure S7: Frequency in which each attribute occurs within the top 5% of best performing models for each species with increasing model complexity (number of attributes in model represented by different coloured lines). Results only include estimates of sown species and only plots with 2+ species sown.

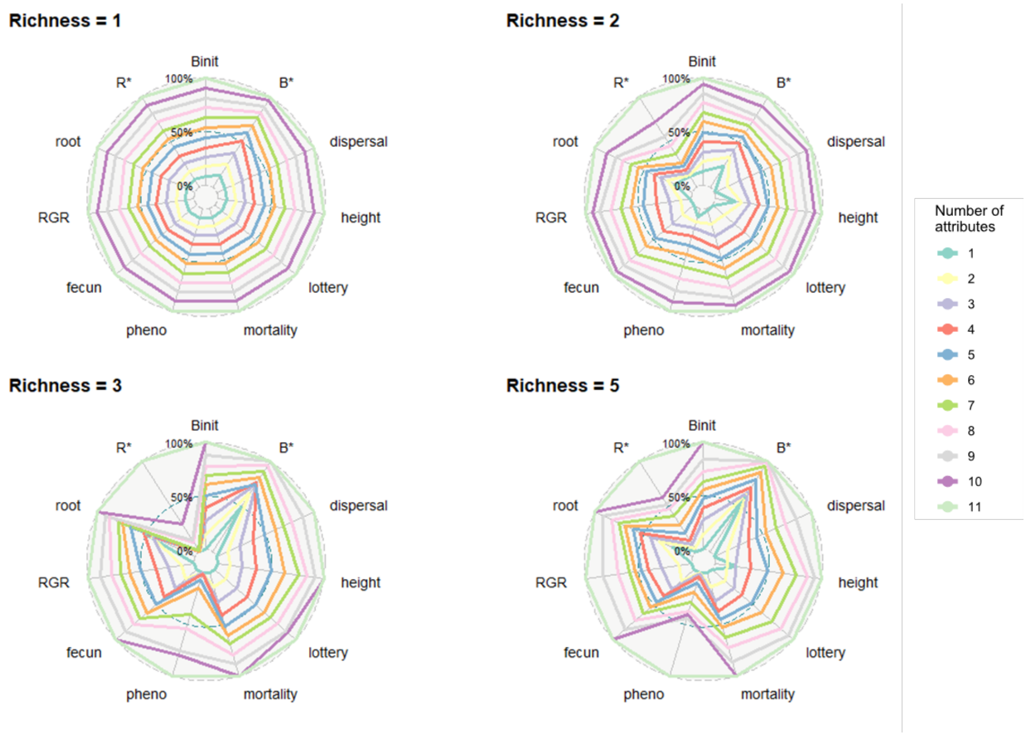

Figure S8: Frequency in which each attribute occurs within the top 5% of best performing models for each sown richness level with increasing model complexity (represented by different coloured lines). Results only include estimates of sown species and only plots with 2+ species sown.

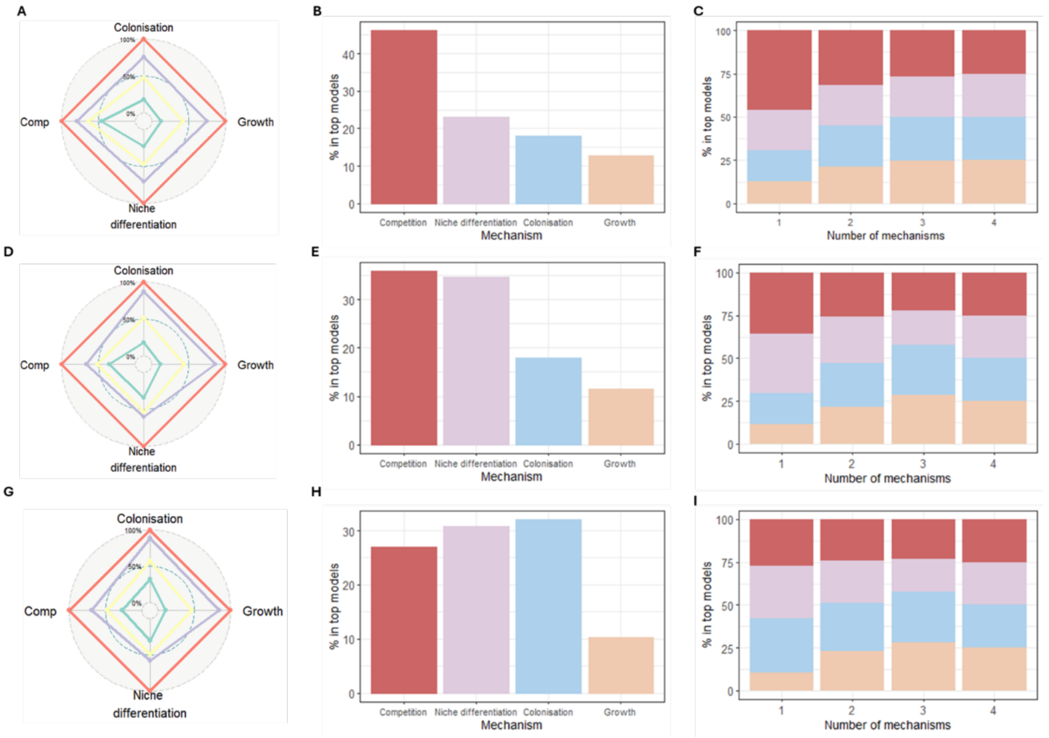

Figure S9: Frequency in which each mechanism occurs within the top 5% of best performing models with a) different number of mechanisms (represented by different coloured lines) or b) a single mechanism included in the model, and c) proportion that each mechanism appears in the top 5% of best performing models for each level of model complexity. Results only show estimates of sown species and in plots with at least two sown species. For a mechanism to be included, it must contain one (upper panels), three (middle panels) or four attributes of each mechanism (lower panels). Where a mechanism contains less than the specified number of attributes (such as growth, which only contains 2 attributes), then it contains all of the attributes.

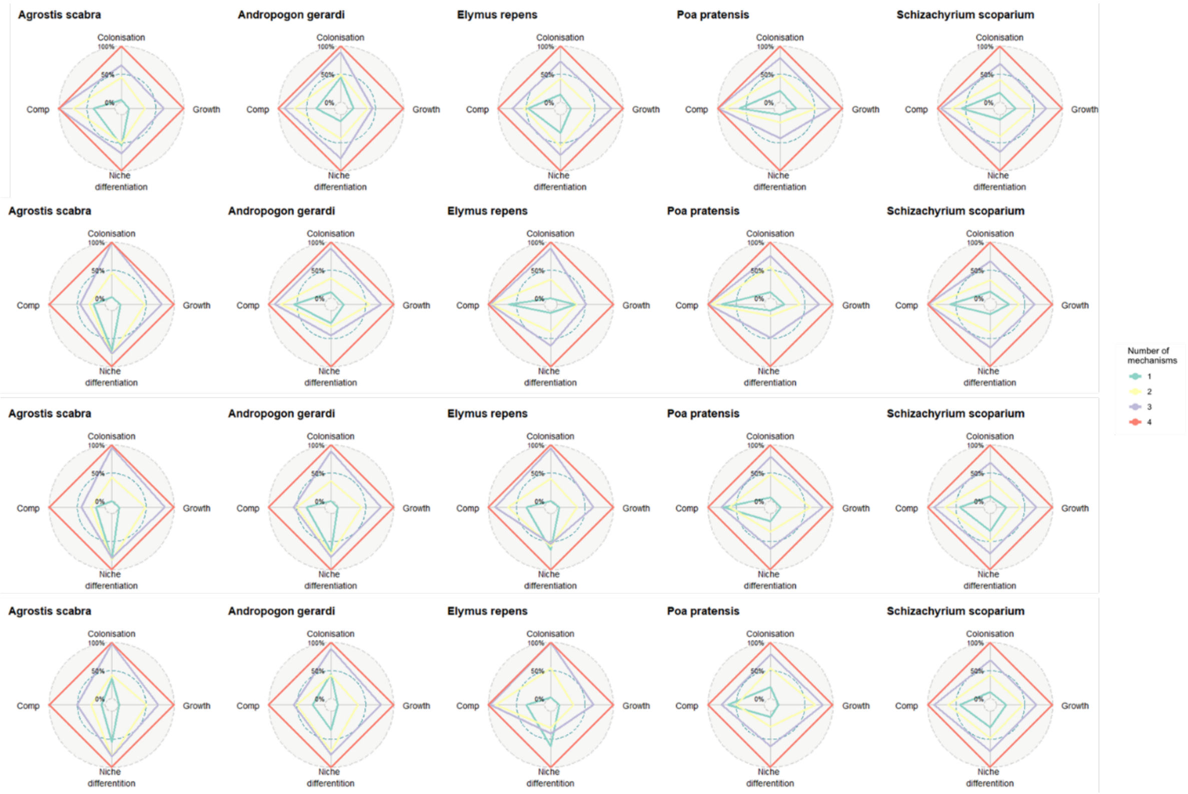

Figure S10: Frequency in which each mechanism occurs within the top 5% of best performing models for each species with different number of groups turned on (represented by different coloured lines). This figure only includes estimates of sown species in plots with two or more sown species. For a mechanism to be included, it must contain one (upper panels), two (second row of panels), three (third row of panels) or four attributes of each mechanism (lower panels). Where a mechanism contains less than the specified number of attributes (such as growth, which only contains 2 attributes), then it contains all of the attributes.

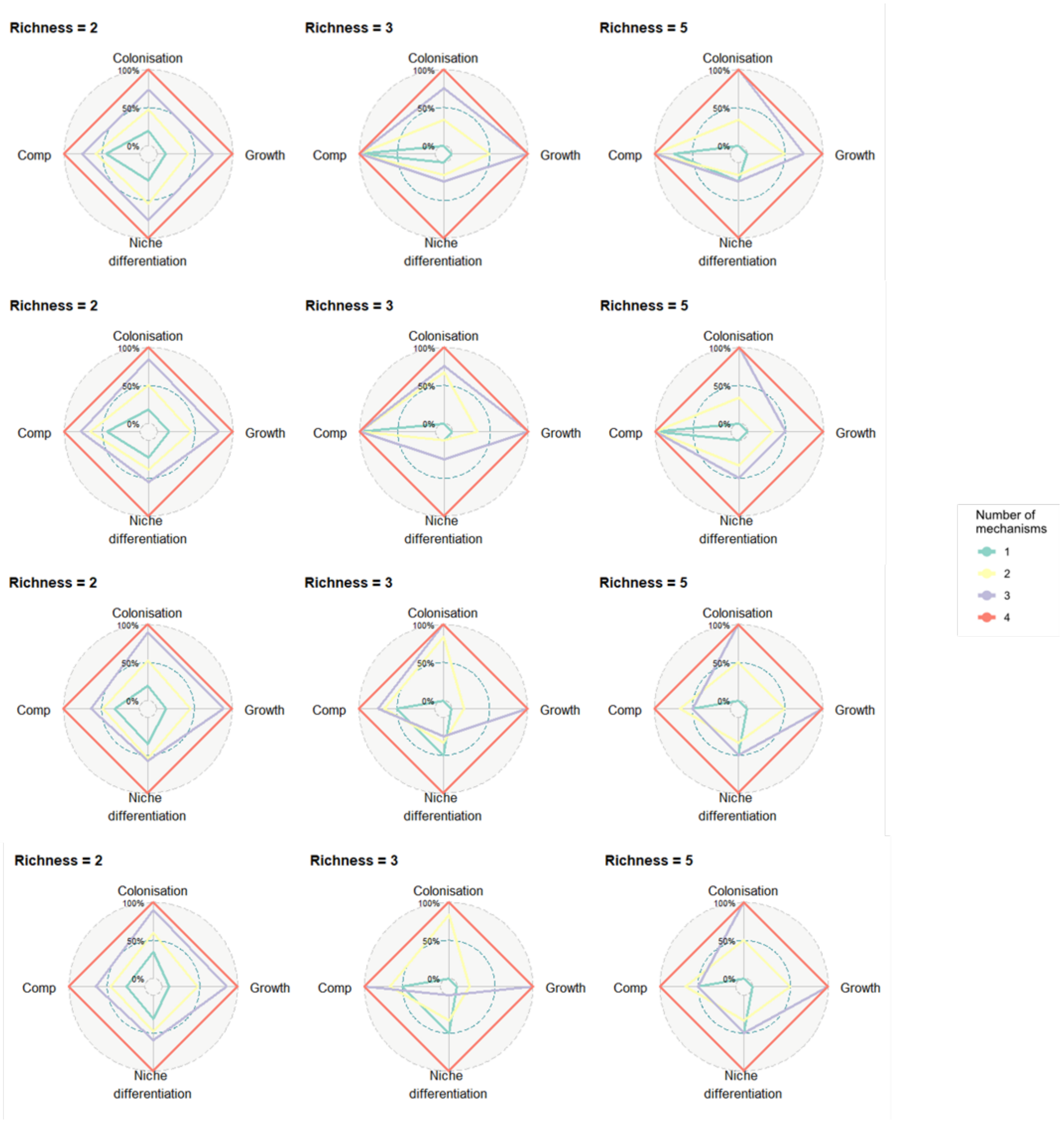

Figure S11: Frequency in which each mechanism occurs within the top 5% of best performing models for each sown richness (columns) with different number of switches turned on (represented by different coloured lines). This plot only includes estimates of sown species. Includes only plots with 2+ species sown. For a mechanism to be included, it must contain one (upper panels), two (second row of panels), three (third row of panels) or four attributes of each mechanism (lower panels). Where a mechanism contains less than the specified number of attributes (such as growth, which only contains 2 attributes), then it contains all of the attributes.

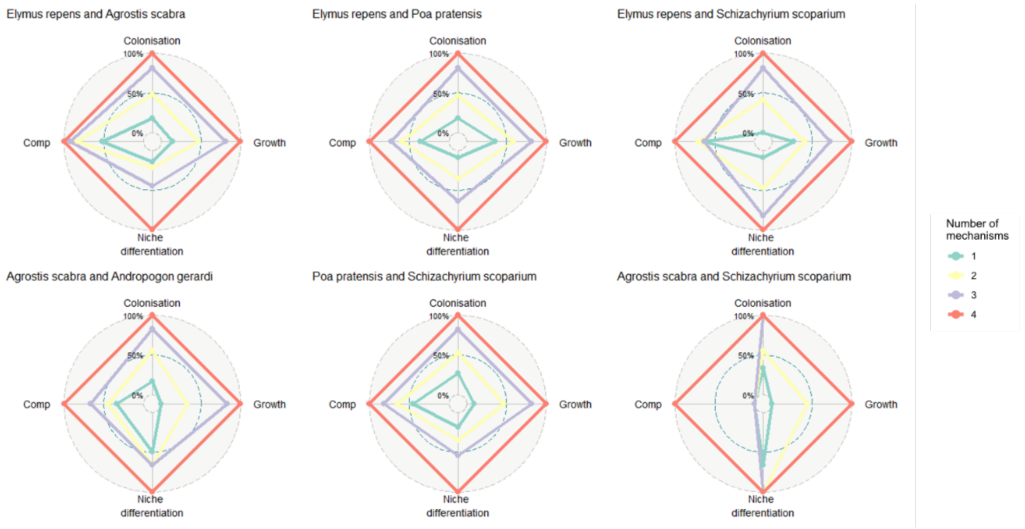

Figure S12: Frequency in which each group occurs within the top 5% of best performing models with different number of groups turned on (represented by different coloured lines) for each 2-species pairwise combination. For a mechanism to be considered included in the model, it must contain *two* attributes from a mechanism.

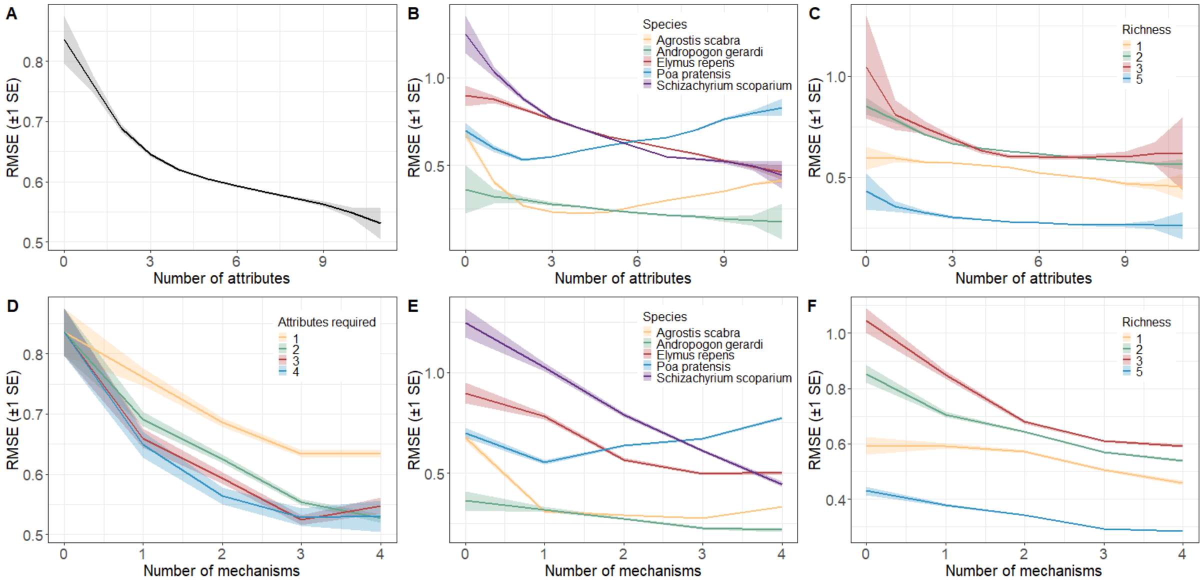

Figure S13: Model error (median root mean square error, RMSE) when different numbers of attributes (upper panels) and mechanisms (lower panels) in the model are switched on.

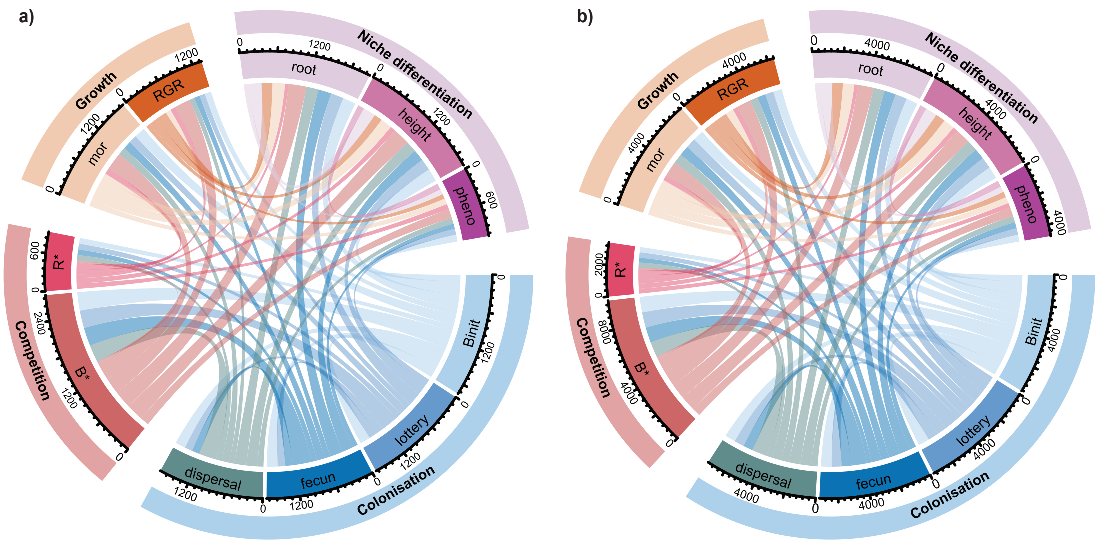

Figure S14: Frequency that attributes co-occur in top-performing models for models with a) three attributes or b) four attributes switched on. The scale in each panel represents the number of times two attribute combinations occur, with a total of 9,579 combinations in panel a, and 38,316 combinations in panel b. The greater number of combinations in panel b is due to the greater number of possible unique attribute combinations when more attributes are included.

Table S1: Parameters used in the multi-mechanism niche model in addition to the 11 attributes that underpin the four ecological mechanism (see Table 1 in main text for information about 11 attributes).

Parameter	Name	Explanation	Data source
**v**	Terminal velocity	Affects dispersal kernel (Wald model), which is an underlying attribute of Colonisation mechanism	Data from Sullivan *et al.* (2018).
**s**	Seed size	Affects lottery function, which is an underlying attribute of Colonisation mechanism	Data from Sullivan *et al.* (2018).
**w**	Mean wind speed throughout growing season	Affects dispersal kernel (Wald model), which is an underlying attribute of Colonisation mechanism<br>Wind speed varies little through the growing season at Cedar Creek. Even when accounting for wind speed at the time each species releases seed, it has little effect on the dispersal kernel so we use mean wind speed	Data from Sullivan *et al.* (2018).
**q**	Soil nitrogen use	Proportion of nitrogen per unit biomass; converts units of biomass to units of nitrogen, which determines how much nitrogen is removed from the soil per g of plant that grows; affects use and biomass that species can reach independently of R*; affects competition	Mean tissue nitrogen concentration, calculated from across all monocultures at Cedar Creek, as reported in Clark *et al.* (2018).
$\boldsymbol{B_{ij}(y,t)}$	Biomass of species *i* in patch *j* on day *t* of the growing season *y*	Species-level biomass on day *t* of the growing season. t(160) is therefore the biomass on the last day of the growing season, before subtracting tissue lost to mortality.	Calculated in model.
$\boldsymbol{b_{ij}(y)}$	Species biomass at beginning of growing season *y*, i.e. $\boldsymbol{b_{ij}(y) = B_{ij}(y,t = 0)}$	Species-level biomass at the start of the year, after accounting for tissue mortality and biomass allocated to reproduction (i.e. fecundity).	Calculated in model.

Table S2: Frequency that attributes co-occur in top-performing models for models with two attributes. Frequency indicates the number of times a pairwise combination occurs, with a total of 1,065 pairwise combinations.

Pair	Attribute 1	Attribute 2	Mechanism 1	Mechanism 2	Frequency	Percentage	Cumulative percentage
1	Binit	B*	Colonization	Competition	49	4.6	4.6
2	dispersal	B*	Colonization	Competition	46	4.3	8.9
3	B*	root	Competition	Differentiation	46	4.3	13.2
4	lottery	B*	Colonization	Competition	45	4.2	17.5
5	fecundity	B*	Colonization	Competition	45	4.2	21.7
6	B*	mortality	Competition	Growth	45	4.2	25.9
7	B*	height	Competition	Differentiation	43	4.0	30.0
8	B*	RGR	Competition	Growth	39	3.7	33.6
9	Binit	root	Colonization	Differentiation	33	3.1	36.7
10	fecundity	root	Colonization	Differentiation	32	3.0	39.7
11	root	height	Differentiation	Differentiation	32	3.0	42.7
12	Binit	height	Colonization	Differentiation	31	2.9	45.6
13	lottery	root	Colonization	Differentiation	31	2.9	48.5
14	dispersal	root	Colonization	Differentiation	30	2.8	51.4
15	mortality	root	Growth	Differentiation	30	2.8	54.2
16	lottery	height	Colonization	Differentiation	29	2.7	56.9
17	fecundity	height	Colonization	Differentiation	27	2.5	59.4
18	mortality	height	Growth	Differentiation	27	2.5	62.0
19	dispersal	height	Colonization	Differentiation	25	2.3	64.3
20	RGR	root	Growth	Differentiation	25	2.3	66.7
21	Binit	fecundity	Colonization	Colonization	22	2.1	68.7
22	B*	phenology	Competition	Differentiation	22	2.1	70.8
23	Binit	mortality	Colonization	Growth	21	2.0	72.8
24	Binit	lottery	Colonization	Colonization	19	1.8	74.6
25	Binit	dispersal	Colonization	Colonization	18	1.7	76.2
26	height	phenology	Differentiation	Differentiation	16	1.5	77.7
27	Binit	R*	Colonization	Competition	15	1.4	79.2
28	lottery	R*	Colonization	Competition	15	1.4	80.6
29	dispersal	R*	Colonization	Competition	15	1.4	82.0
30	Binit	phenology	Colonization	Differentiation	15	1.4	83.4
31	R*	mortality	Competition	Growth	15	1.4	84.8

32	R*	height	Competition	Differentiation	14	1.3	86.1
33	R*	phenology	Competition	Differentiation	14	1.3	87.4
34	R*	RGR	Competition	Growth	14	1.3	88.7
35	fecundity	R*	Colonization	Competition	13	1.2	90.0
36	lottery	phenology	Colonization	Differentiation	13	1.2	91.2
37	dispersal	phenology	Colonization	Differentiation	12	1.1	92.3
38	mortality	phenology	Growth	Differentiation	12	1.1	93.4
39	root	phenology	Differentiation	Differentiation	11	1.0	94.5
40	RGR	height	Growth	Differentiation	11	1.0	95.5
41	R*	root	Competition	Differentiation	10	0.9	96.4
42	fecundity	phenology	Colonization	Differentiation	8	0.8	97.2
43	RGR	phenology	Growth	Differentiation	6	0.6	97.7
44	B*	R*	Competition	Competition	5	0.5	98.2
45	Binit	RGR	Colonization	Growth	3	0.3	98.5
46	lottery	RGR	Colonization	Growth	3	0.3	98.8
47	fecundity	RGR	Colonization	Growth	3	0.3	99.1
48	mortality	RGR	Growth	Growth	3	0.3	99.3
49	lottery	mortality	Colonization	Growth	2	0.2	99.5
50	dispersal	mortality	Colonization	Growth	2	0.2	99.7
51	dispersal	RGR	Colonization	Growth	2	0.2	99.9
52	fecundity	mortality	Colonization	Growth	1	0.1	100.0
53	lottery	fecundity	Colonization	Colonization	0	0.0	100.0
54	lottery	dispersal	Colonization	Colonization	0	0.0	100.0
55	fecundity	dispersal	Colonization	Colonization	0	0.0	100.0

Table S3: Pearson correlation coefficients (r) among model attributes, grouped by mechanism. **r** > |0.7| in bold font. When an attribute was not at species level (root, height, phenology, Binit), correlations were taken from the species average across all pairwise comparisons and experimental treatments (Table S5). We do not present correlations for lottery and dispersal (attributes of Colonization) as both are functions in the model rather than values. Mean absolute correlations show mean correlation (|r|) among attributes from the same mechanism and from different mechanisms. Colours highlight within-mechanism correlations.

**Mechanism – Attribute**	Competition – B*	Competition – R*	Growth – Mortality	Growth – Growth rate	Differentiation – Root	Differentiation – Height	Differentiation – Phenology	Colonization – Initial biomass	Colonization – Fecundity
Competition – R*	-0.55								
Growth – Mortality	-0.39	**0.70**							
Growth – Growth rate	-0.54	0.54	**0.81**						
Differentiation – Root	**-0.86**	**0.71**	0.66	0.50					
Differentiation – Height	**-0.85**	0.48	**0.71**	**0.82**	**0.82**				
Differentiation – Phenology	0.50	-0.05	-0.46	-0.27	-0.69	-0.70			
Colonization – Initial biomass	-0.09	-0.44	**-0.84**	-0.43	-0.36	-0.31	0.42		
Colonization – Fecundity	0.39	-0.06	0.58	0.48	-0.16	0.15	-0.17	**-0.74**	
**Mean absolute correlations (|r|)**									
Within mechanism	0.55	0.55	**0.81**	**0.81**	**0.75**	**0.76**	**0.70**	**0.74**	**0.74**
Between mechanism	0.51	0.43	0.62	0.51	0.54	0.55	0.31	0.41	0.28

Table S4: Trait values of five grass species sown in the empirical experiment. All values taken from populations grown in field conditions across Cedar Creek except for data about: i) monocultures biomass, which was the mean biomass from year 6 through year 9 (4 years of data) from e026 (excluding block 5, which was fertilised); and ii) *Andropogon* and *Schizachylrium* mortality, which were from Lauenroth and Adler (2008).

Species	Monoculture biomass (g/m^2; "B*")	Tissue N fraction (g N/g biomass; "q")	R* (mg nitrate / kg soil; "R*")	First month of growing season ("PhenStart")	Last month of growing season ("PhenEnd")	Height (m, "h")	Rooting depth (m, "rd")	Mortality (fraction per year, "m")	Relative growth rate per day ("RGR")	Fecundity (% above ground biomass to seed, "f")	Seed size (g; "s")	Terminal velocity (m/s; v)
Agrostis scabra	48.6	0.013	0.156	5	9	0.39	0.32	0.16	0.076	0.115	0.00006	1.14
Elymus repens	24.3	0.015	0.135	5	10	0.63	0.20	0.05	0.035	0.005	0.00162	1.56
Poa pratensis	33.0	0.014	0.103	3	11	0.19	0.08	0.14	0.054	0.095	0.00024	1.24
Andropogon gerardi	107.3	0.009	0.066	7	10	1.40	3.00	0.03	0.017	0.087	0.00154	1.60
Schizachyrium scoparium	54.1	0.007	0.047	5	10	0.61	1.75	0.03	0.049	0.073	0.00113	1.37

Table S5: Summary of 43 seed sowing treatments used in empirical grassland experiment (e026). Species: Er = *Elymus repens*; Pp = *Poa pratensis*; Ss = *Schizachyrium scoparium*; Er_CCESR = *Elymus repens* (seed collected from populations at Cedar Creek Ecosystem Science Reserve); As = *Agrostis scabra*; Ag = *Andropogon gerardii*. Type refers to seed introduction sequence: mono = monocultures; Dewit = Plots are seeded at the same time with the ratio of the two species changing while the total density remains constant; SeedInv = Seed of a second species is sown into a high density stand of the first species, which has grown for a year (seed sowing year indicated by y1 and y2); Est.Inv. = Each subplot is divided into four units, two of which are planted at high density with one species, two with the other species. After the first season the barriers separating the four units will be removed; Mult.Sp. = Multiple species are sown into plots at the same time with an equal ratio across all sown species such that the total density remains constant. Number of replicate plots for each treatment in each of ten blocks are shown (total of 64 plots in each block).

Treatment	Species	Type	Total density (seedlings/m^2)	Seed sowing ratio	Replicates per block
**1**	Er	mono	3000	1	4
**2**	Pp	mono	3000	1	3
**3**	Ss	mono	3000	1	3
**4**	Er_CCESR	mono	3000	1	1
**5**	As	mono	3000	1	4
**6**	As	mono	600	1	2
**7**	Ag	mono	3000	1	2
**8**	Er	mono	600	1	2
**9**	Pp, Ss	Dewit	3000	0.5: 0.5	1
**10**	Pp, Ss	Dewit	3000	0.2: 0.8	1
**11**	Pp, Ss	Dewit	3000	0.8: 0.2	1
**12**	Ss, Er	Dewit	3000	0.5: 0.5	1
**13**	Ss, Er	Dewit	3000	0.2: 0.8	1
**14**	Ss, Er	Dewit	3000	0.8: 0.2	1
**15**	Er, Pp	Dewit	3000	0.5: 0.5	1
**16**	Er, Pp	Dewit	3000	0.2: 0.8	1
**17**	Er, Pp	Dewit	3000	0.8: 0.2	1
**18**	As, Er	Dewit	3000	0.5: 0.5	3

Treatment	Species	Type	Total density (seedlings/m^2)	Seed sowing ratio	Replicates per block
**19**	As, Er	Dewit	3000	0.2: 0.8	3
**20**	As, Er	Dewit	3000	0.8: 0.2	3
**21**	As, Er	Dewit	600	0.5: 0.5	1
**22**	As, Er	Dewit	600	0.2: 0.8	1
**23**	As, Er	Dewit	600	0.8: 0.2	1
**24**	As, Ag	Dewit	3000	0.5: 0.5	1
**25**	As, Ag	Dewit	3000	0.2: 0.8	1
**26**	As, Ag	Dewit	3000	0.8: 0.2	1
**27**	As, Ss	Dewit	3000	0.5: 0.5	1
**28**	As, Ss	Dewit	3000	0.2: 0.8	1
**29**	As, Ss	Dewit	3000	0.8: 0.2	1
**30**	Pp, Ss	SeedInv.	6000	0.5(y1): 0.5(y2)	1
**31**	Pp, Er	SeedInv.	6000	0.5(y1): 0.5(y2)	1
**32**	Ss, Pp	SeedInv.	6000	0.5(y1): 0.5(y2)	1
**33**	Ss, Er	SeedInv.	6000	0.5(y1): 0.5(y2)	1
**34**	Er, Pp	SeedInv.	6000	0.5(y1): 0.5(y2)	1
**35**	Er, Ss	SeedInv.	6000	0.5(y1): 0.5(y2)	1
**36**	As, Er	SeedInv.	6000	0.5(y1): 0.5(y2)	1
**37**	Er, As	SeedInv.	6000	0.5(y1): 0.5(y2)	1
**38**	Er, Pp	Est.Inv.	3000	0.5: 0.5	1
**39**	Er, Ss	Est.Inv.	3000	0.5: 0.5	1
**40**	Pp, Ss	Est.Inv.	3000	0.5: 0.5	1
**41**	As, Er	Est.Inv.	3000	0.5: 0.5	1
**42**	Pp, Er, Ss	Mult.Sp.	3000	1/3: 1/3: 1/3	2
**43**	As, Er, Ag, Ss, Pp	Mult.Sp.	3000	0.2: 0.2: 0.2: 0.2: 0.2	2

Table S6: Pearson correlation coefficients among trait values of five study species. **r** > |0.7| in bold font.

	B*	Height	Mortality	PhenEnd	PhenStart	Tissue N	R*	Fecundity	Growth rate	Root depth	Seed mass
Height	**0.87**										
Mortality	-0.39	-0.69									
PhenEnd	-0.17	-0.16	-0.16								
PhenStart	**0.81**	**0.93**	-0.57	-0.50							
Tissue N	**-0.71**	-0.54	0.57	0.04	-0.51						
R*	-0.55	-0.48	0.70	-0.41	-0.29	**0.88**					
Fecundity	0.39	-0.12	0.58	-0.16	-0.07	-0.31	-0.06				
Growth rate	-0.54	**-0.82**	**0.81**	-0.35	-0.59	0.32	0.54	0.48			
Root depth	**0.93**	**0.89**	-0.67	-0.07	**0.81**	**-0.85**	**-0.76**	0.16	-0.67		
Seed mass	0.33	**0.73**	**-0.93**	0.08	0.63	-0.29	-0.44	**-0.73**	**-0.88**	0.55	
Terminal velocity	0.41	**0.79**	**-0.86**	0.17	0.64	-0.22	-0.40	-0.67	**-0.95**	0.57	**0.97**

SI References

Clark, A.T., Lehman, C. & Tilman, D. (2018). Identifying mechanisms that structure ecological communities by snapping model parameters to empirically observed tradeoffs. *Ecol. Lett.*, 21, 494-505.

Global Invasive Species Database (2025). Species profile: *Poa pratensis*. Available at: http://www.iucngisd.org/gisd/species.php?sc=1419 Last accessed 9 January 2025.

Lauenroth, W.K. & Adler, P.B. (2008). Demography of perennial grassland plants: survival, life expectancy and life span. *J. Ecol.*, 96, 1023-1032.

Matthews, R.F. (1992). *Agrostis scabra*. Available at: https://www.fs.usda.gov/database/feis/plants/graminoid/agrsca/all.html Last accessed January 9 2025.

Snyder, S.A. (1992). *Elymus repens*. Available at: https://www.fs.usda.gov/database/feis/plants/graminoid/elyrep/all.html Last accessed January 9 2025.

Steinberg, P.D. (2002). *Schizachyrium scoparium*. Available at: https://www.fs.usda.gov/database/feis/plants/graminoid/schsco/all.html Last accessed 9 January 2025 2025.

Sullivan, L.L., Clark, A.T., Tilman, D. & Shaw, A.K. (2018). Mechanistically derived dispersal kernels explain species-level patterns of recruitment and succession. *Ecology*, 99, 2415-2420.

Tercek, M.T. & Whitbeck, J.L. (2004). Heat avoidance life history strategy controls the distribution of geothermal Agrostis in Yellowstone. *Ecology*, 85, 1955-1966.

Tilman, D. & Cowan, M.L. (1989). Growth of old field herbs on a nitrogen gradient. *Funct. Ecol.*, 3, 425-438.

Tilman, D. & Wedin, D. (1991a). Dynamics of nitrogen competition between successional grasses. *Ecology*, 72, 1038-1049.

Tilman, D. & Wedin, D. (1991b). Plant traits and resource reduction for five grasses growing on a nitrogen gradient. *Ecology*, 72, 685-700.

Uchytil, R.J. (1988). *Andropogon gerardii*. Available at: https://www.fs.usda.gov/database/feis/plants/graminoid/andger/all.html Last accessed January 9 2025.

Uchytil, R.J. (1993). *Poa pratensis*. Available at: https://www.fs.usda.gov/database/feis/plants/graminoid/poapra/all.html Last accessed January 9 2025.

Weaver, J.E. (1926). *Root Development of Field Crops*. 1 edn. McGraw-Hill Book Company, Inc. , New York.

Wedin, D. & Tilman, D. (1993). Competition among grasses along a nitrogen gradient: initial conditions and mechanisms of competition. *Ecol. Monogr.*, 63, 199-229.
